# Quantifying evolving toxicity in the TAML/peroxide mineralization of propranolol

**DOI:** 10.1016/j.isci.2020.101897

**Published:** 2020-12-07

**Authors:** Yogesh Somasundar, Abigail E. Burton, Matthew R. Mills, David Z. Zhang, Alexander D. Ryabov, Terrence J. Collins

**Affiliations:** 1 Institute for Green Science, Department of Chemistry, Carnegie Mellon University, 4400 Fifth Avenue, Pittsburgh, PA 15213, USA

**Keywords:** Chemical Engineering, Environmental Chemical Engineering, Green Chemistry, Environmental Chemistry

## Abstract

Oxidative water purification of micropollutants (MPs) can proceed via toxic intermediates calling for procedures for connecting degrading chemical mixtures to evolving toxicity. Herein, we introduce a method for projecting evolving toxicity onto composite changing pollutant and intermediate concentrations illustrated through the TAML/H_2_O_2_ mineralization of the common drug and MP, propranolol. The approach consists of identifying the key intermediates along the decomposition pathway (UPLC/GCMS/NMR/UV-Vis), determining for each by simulation and experiment the rate constants for both catalytic and noncatalytic oxidations and converting the resulting predicted concentration versus time profiles to evolving composite toxicity exemplified using zebrafish lethality data. For propranolol, toxicity grows substantially from the outset, even after propranolol is undetectable, echoing that intermediate chemical and toxicity behaviors are key elements of the environmental safety of MP degradation processes. As TAML/H_2_O_2_ mimics mechanistically the main steps of peroxidase catalytic cycles, the findings may be relevant to propranolol degradation in environmental waters.

## Highlights

• TAML(s) catalyzed H_2_O_2_ mineralization of persistent micropollutant propranolol
• Major intermediates kinetically characterized for catalytic and noncatalytic reactions
• Method introduced quantifying evolving composition and toxicity of process solution
• Toxicity grew after complete propranolol removal—toxicity data gap highlighted

## Graphical Abstract

**Figure:**
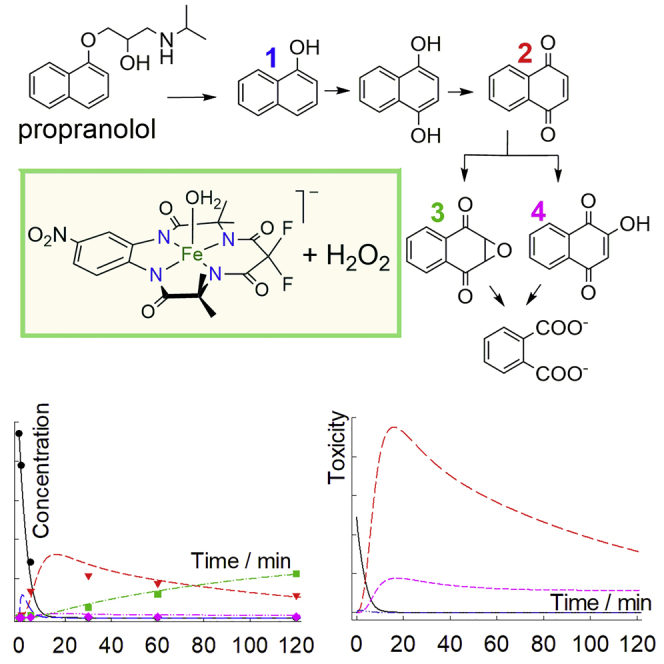

## Introduction

Oxidation catalysis via monooxygenase and peroxidase enzymes provides Nature's most commonly deployed and most potent tools for decomposing toxic organic compounds. Typically, those that react too slowly with these enzymes turn up as persistent pollutants in water. Since Rachel Carson's 1962 *Silent Spring* (Carson, 2002), a key water purification field for sustainability has awaited the design of synthetic catalysts that can activate natural oxidants such as H_2_O_2_ to effectively destroy persistent organic pollutants in scalable ways, cleaning water the way that Nature does, only better. The multi-faceted challenges encompass the extremely difficult removal from water of traces of chemicals that produce adverse effects at low doses or concentrations (low ppt–low ppb) called micropollutants (MPs) (Schug et al., 2013). MPs have been identified as natural and artificial estrogens and other hormones, drugs, pesticides, dioxins and polychlorinated biphenyls (PCBs), labile polymer monomers such as BPA, polymer additives such as BPA and phthalates, antimicrobials, fire retardants, polycyclic aromatic hydrocarbons (PAHs), and many others. The need for viable biomimicking catalytic solutions that, under ambient conditions, can safely treat vast quantities of wastewaters contaminated by ultradilute (≤2 ppb) MPs demands that oxidation catalysts exhibit not only unprecedented technical and cost performances, but also high health, environmental, and fairness performances (Somasundar, 2020; Warner et al., 2019). As oxidative pollutant degradations can pass through potentially toxic intermediates that may be even more persistent and/or toxic than the starting MP before arriving at the safety of near-mineral to mineral endpoints, it is vital to understand the relative toxicities of all components in the degradation sequence and the composite evolving toxicity from the beginning to the end of a degradation process.

**Figure 1:**
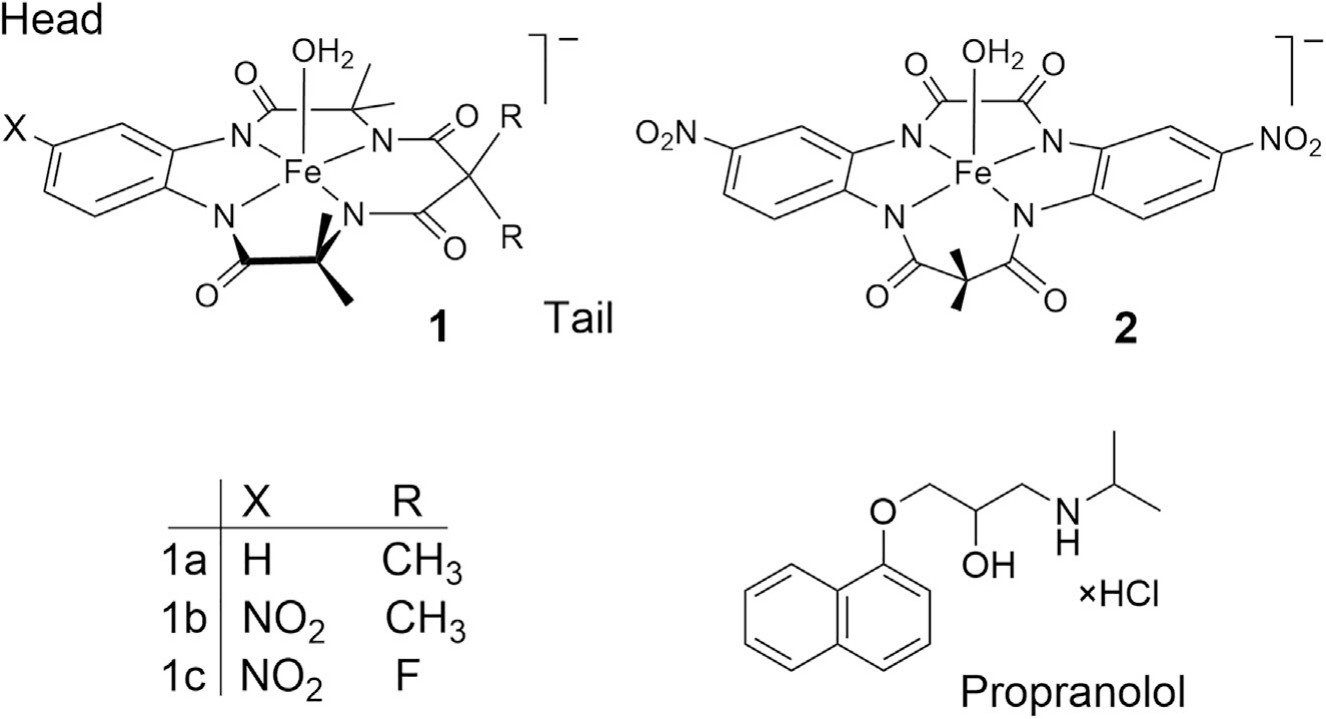
Propranolol and TAML activators 1 and 2 used in this study

A conceivable solution for obtaining this information is described in this work by the example of a detailed TAML/peroxide degradation study of the widely used β-blocker drug, propranolol (1-isopropylamino-3-(naphthalen-1-yloxy)propan-2-ol, Figure 1). (Keelan, 1965; Prichard, 1964; Stapleton, 1997) Propranolol is a commonly found MP in water. In our execution of this study, propranolol is oxidized to near mineralization by TAML/H_2_O_2_. Six larger aromatic intermediates have been identified and characterized among which 1,4-naphthoquinone **C** (Scheme 2) could be isolated. Then, a computer simulation was developed with KinTek Explorer for the total **1c**/H_2_O_2_ propranolol degradation. The simulation affords rate constants for degradation of each intermediate in the TAML-catalyzed reaction, *k*_II,S_, and also for uncatalyzed degradation steps, *k*_2,S_, which have been validated by direct kinetic studies of these intermediates via the kinetic methods described in a previous article(Somasundar et al., 2018). The experimental rate constants *k*_II_ (for the TAML-catalyzed reaction) and *k*_2_ (for uncatalyzed reactions) are shown to agree well with the simulated *k*_II,S_ and *k*_2,S_ values. Because of the competitive nature of the kinetics in degrading mixtures, this close match gives us confidence that the major aromatic intermediates had all been identified. Next, with the concentrations of all key components at any given time revealed, the evolution of acute toxicity toward zebrafish embryos has been estimated for the total **1c**/H_2_O_2_ propranolol degradation from known toxicities for the individual species (one surrogate) in the degradation sequence.

**Scheme 1:**
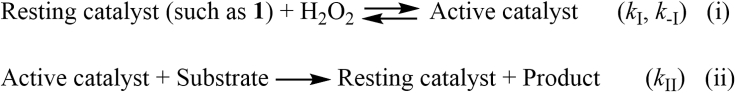
Typical stoichiometric mechanism of oxidative catalysis by TAML activators

Our oxidation system is composed of hydrogen peroxide as the primary oxidant and an iron(III)-TAML peroxide activator (Figure 1), that closely replicates key steps of the catalytic cycles of peroxidase and cytochrome P450 enzymes (Collins, 2002; Collins et al., 2010, Collins and Ryabov, 2017; Ryabov and Collins, 2009). In water, TAMLs catalyze the oxidation of a broad spectrum of molecules, including MPs (Kundu et al., 2013; Mills et al., 2015; Somasundar et al., 2020; Tang et al., 2016; Warner et al., 2020) according to Scheme 1. The mechanism in Scheme 1, when the oxidation is not complicated by extra phenomena (Somasundar et al., 2018), leads to the kinetic Equation 1. The rate constant *k*_-I_ is usually negligible (Chahbane et al., 2007).

(Equation 1) $$-\frac{d\left[S\right]}{dt}=\frac{k_{I}k_{II}\left[H_{2}O_{2}\right]\left[S\right]}{k_{-I}+k_{I}\left[H_{2}O_{2}\right]+k_{II}\left[S\right]}\left[Fe_{t}^{\text{III}}\right]$$

Recently, the catalytic cycle of the TAML/H_2_O_2_ propranolol oxidation was examined in considerable kinetic depth where the entire focus was on detecting and quantifying all interactions of propranolol with TAML species (Somasundar et al., 2018). The different focus of this contribution is on the identification of propranolol oxidation intermediates, their kinetic characterization, and the building and interrelating of the evolving mass and toxicity profiles in the total propranolol degradation.

## Results and discussion

### UPLC and GC-MS identification of fragments of TAML-catalyzed oxidation of propranolol by H_2_O_2_

**Figure 2:**
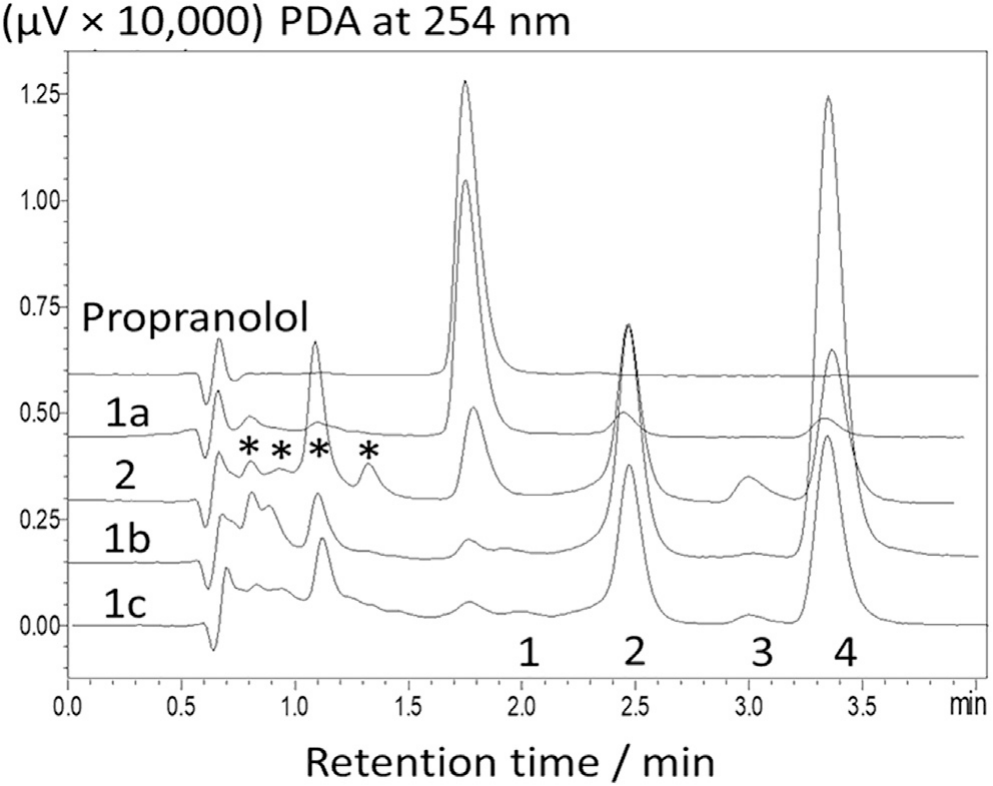
UPLC chromatograms of the products of propranolol degradation by TAML/H_2_O_2_ UPLC chromatograms identified at 254 nm using a photo-diode array detector. Conditions: [TAML] = 1 × 10^−6^ M, [propranolol] = 50 × 10^−6^ M, [H_2_O_2_] = 5 × 10^−3^ M, pH 7 (0.01 M phosphate), 25°C, reaction time 2 h. The numbered peaks derive from the following: 1 **F**, 2 **D**, 3 **E**, 4 **C** (see text). See also Figures S1–S7.

The **1c**/H_2_O_2_ system is particularly efficient in degrading propranolol where a 1 μM loading of **1c** with 5 mM H_2_O_2_ will digest all propranolol (50 μM) at pH 7 (30 min) and 9 (2 min), respectively, as confirmed by ultrahigh pressure liquid chromatography (UPLC) with fluorescence detection (Somasundar et al., 2018). No intermediates were detectable with this technique. However, when the chromatograms were analyzed with photo-diode array detection (254 nm, Figure 2), several degradation products with retention times exceeding that of propranolol (1.8 min) were observed. For all four TAML activators studied (**1a**, **b**, and **c,** and **2**) identical intermediates are generated, the amounts of which at any given time vary as expected in proportion to the diverse catalytic activity of the various TAMLs toward both propranolol and its degradation products. Considerably less intermediate material was observed at 2 h with **1c**, the most effective TAML used (Figure 2), and no intermediates were observed for the **1c**/H_2_O_2_ process after 8 h.

The chromatograms also exhibit peaks with retention times <1.8 min (indicated by asterisks, Figure 2). Corresponding compounds eluted almost immediately suggesting that they are more polar than propranolol, perhaps smaller organic molecules, as products of the deep fragmentation of propranolol. The ionic chromatography data presented below support this hypothesis. Peaks 1‒4 in Figure 2 arise from compounds less polar than propranolol, and their assignment became our premier characterization goal. Several approaches have been applied by other workers who have identified propranolol oxidative degradation products before our work with TAML/peroxide (Anquandah et al., 2013; Benner and Ternes, 2009; Ganiyu et al., 2017; Isarain-Chavez et al., 2011; Quintana et al., 2012); Santiago-Morales et al. (2013) This literature shows that different advanced oxidation processes can return different oxidation products. For example, Benner et al., determined that the primary ozonation product of propranolol transformation was a dialdehyde (m/z = 292) obtained from naphthalene ring opening (Benner and Ternes, 2009). This dialdehyde was further hydroxylated at the secondary amine N to produce an oxidation product with m/z = 308, which upon ozone attack loses a glyoxal moiety to provide another major transformation product with m/z = 282. Interestingly, similar major oxidation products were also observed for ferrate(VI) oxidation of propranolol by Anquandah et al., suggesting a similar mechanistic pathway to ozone (Anquandah et al., 2013). For electro-Fenton and photoelectro-Fenton reactions, authors Isarain-Chavez et al., identified 1-naphthol, 1,4-naphthoquinone and phthalic acid as some of the major transformation products (Isarain-Chavez et al., 2011). The authors further observed the formation of acetic acid, oxalic acid, oxamic acid, and nitrates en route to propranolol mineralization. These published works with their respective transformation products have informed our deep dive into the temporal dynamics of the **1c**/H_2_O_2_ oxidation of propranolol.

The changing areas of peaks 1‒4 and the propranolol peak were monitored over 8 h and plotted versus time. Primary data that are consistent across all four TAMLs are illustrated for **1c** (Figure S1) indicating that peak 4 appears first and its associated compound is then further oxidized to peaks 1‒3 compounds. The maximum amount of the peak 4 compound is achieved at ca. 30 min, which then decreases with continued catalysis (Figure S1). Note that all peaks disappear after 8 h of **1c**/H_2_O_2_ oxidative treatment.

Peak 4 is assigned to 1,4-naphthoquinone **C,** which was isolated using larger initial loadings of both propranolol and **1c**, with addition of H_2_O_2_ in multiple aliquots over 24 h to minimize the catalase-like activity of **1c** (Ghosh et al., 2008). The isolated and purified material was characterized by ^1^H NMR and mass spectrometry (Figure S2) and tied to peak 4 (Figure 2) by running the reaction at a low [H_2_O_2_] to achieve only peak 4, followed by UPLC analysis of this solution with standard 1,4-naphthoquinone spiking (spiking experiment) (Figure S3). Correspondingly, peak 1‒3 compounds derive from 1,4-naphthoquinone; they were identified by gas chromatography-mass spectrometry (GC-MS) or IC and confirmed by UPLC spiking experiments.

The gas chromatogram of the reaction products after solid-phase extraction into methanol contains two high-intensity peaks and several peaks of much lower intensity (Figure S4). The major two correspond to unreacted propranolol (16.57 min) and 2,3-dihydro-2,3-epoxynaphthalene-1,4-dione (**D**, 12.92 min, Figure S5; confirmed by matching with published MS spectra, Nonoyama et al., 2001). As a product of 1,4-naphthoquinone oxidation, **D** could give rise to one of 1‒3 peaks in Figure 2—spiking associated **D** with peak 2 (Figure S3). The nature of peak 3 was determined by GC-MS analysis (Figure S4) of the methanol extract. A small peak at 12.32 min corresponds to already identified 1,4-naphthoquinone **C;** its GC-MS spectrum was indistinguishable from that in Figure S2C. The 13.08-min peak originates from 2-hydroxynaphthalene-1,4-dione **E** as confirmed by GC-MS with standard matching (Figure S6). Spiking (Figure S3) associated **E** with peak 3 (Figure 2). GC-MS also identified 1-naphthol **A** as a propranolol degradation product (12.75-min peak in Figure S4) by comparison with a spectrum of authentic 1-naphthol (Figure S7). Peak 1 in Figure 2 belongs to phthalic acid **F** (spiking), a common product of oxidative degradation of propranolol identified by many workers (Anquandah et al., 2013; Isarain-Chavez et al., 2011).

### Ionic chromatographic identification of fragments of TAML-catalyzed oxidation of propranolol by H_2_O_2_

**Figure 3:**
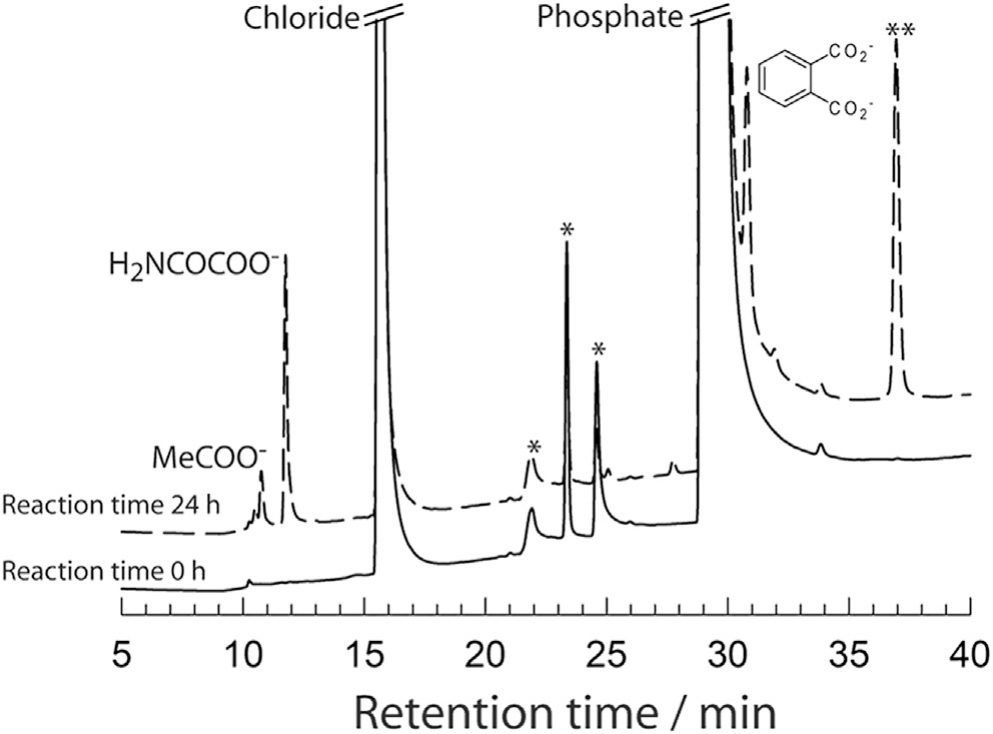
Ionic chromatogram showing products of propranolol degradation by **1c**/H_2_O_2_ Ionic chromatograms identified using a conductivity detector. Conditions: [**1c**] = 1 × 10^−6^ M, [propranolol] = 500 × 10^−6^ M, [H_2_O_2_] = 5 × 10^−3^ M, pH 7 (0.01 M phosphate), 25°C, reaction time 24 h. See also Figures S8.

Phthalic acid and small organic acids were also confirmed by ion chromatography (Figure 3). Acetate (10.8 min), oxamate (11.7 min), and phthalate (30.8 min) were reliably identified by running standards separately. Peak (∗∗) (~36.9 min) was not identified. Phosphate (29 min) from the buffer and chloride (15.6 min) from the propranolol hydrochloride were also detected. Peaks marked with asterisks are due to contaminants in the original propranolol sample.

### ^**1**^H NMR identification of acetone

TAML-catalyzed degradation of organic compounds by H_2_O_2_ often produces acetone as in the case of bisphenol A (Onundi et al., 2017). Acetone was detected as a propranolol degradation product by ^1^H NMR in D_2_O at 2.26 ppm (literature value of 2.22 ppm, Gottlieb et al., 1997) (Figure S8).

**Figure 4:**
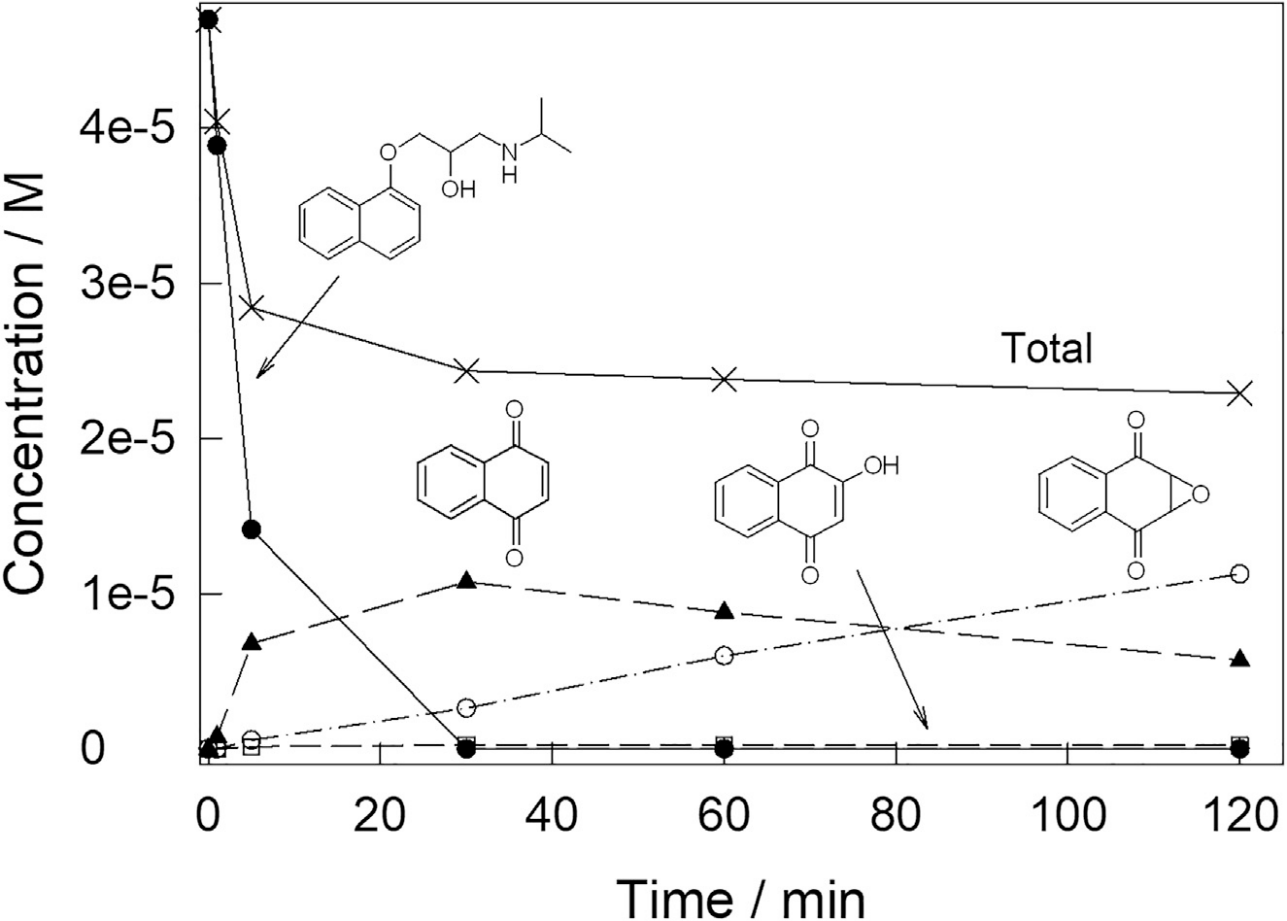
Mass (concentration) profiles for propranolol and its intermediates Concentration versus time profiles for propranolol and the UPLC observable products of its **1c**/H_2_O_2_ degradation (lines are for emphasis only). The total data sum the concentrations of all organic material in the graph. Conditions: [**1c**] 1 × 10^−6^ M, [propranolol] 50 × 10^−6^ M, [H_2_O_2_] 5 × 10^−3^ M pH 7 (0.01 M phosphate), 25°C.

The mineralization of propranolol requires 43 equiv of H_2_O_2_ as noted in the first part of this work (Somasundar et al., 2018). Herein, we utilized 100 equiv of H_2_O_2_ (50 × 10^−6^ M propranolol, 5 × 10^−3^ M H_2_O_2_), more than enough to effect mineralization given that our catalytic processes do not waste peroxide. We tracked the progress of propranolol and its observable transformation products over time (Figures 4 and S1). Propranolol and all its degradation intermediates are undetectable after 8 h (Figure S1). However, by ion chromatography small organic acid fragments were observed: phthalic, acetic, and oxamic acids. Acetone was detected by ^1^H NMR. These analyses following the fate of the carbon are more powerful than commonly used total organic carbon analyses. Based on the data presented, we can convincingly state that all propranolol was nearly mineralized and most of it was mineralized.

### Building concentration versus time profiles for the “UPLC” products of 1c/H_2_O_2_ propranolol destruction

**Scheme 2:**
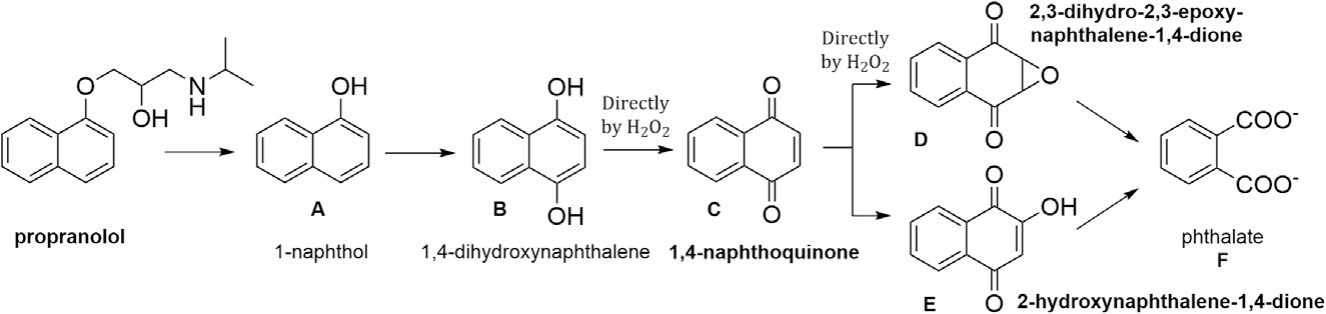
Sequence of oxidation of the naphthalene moiety of propranolol Chain of events associated with **1c**/H_2_O_2_ oxidative degradation of the naphthalene unit of propranolol at pH 7 and 25°C. Quantified by UPLC compounds (**C**-**E**) are set in bold; 1-naphthol **A** was identified by GC-MS. 1,4-Dihydroxynaphthalene **B** is a postulated intermediate separating 1-naphthol **A** and 1,4-naphthoquinone **C**—see text for details.

Scheme 2 describes what is clearly the major route in propranolol degradation by **1c**/H_2_O_2_. Having identified peaks 1‒4, the qualitative concentration profiles in Figure S1 were quantified using UPLC calibration curves for propranolol, **C**, **D**, and **E** (limit of detection [LOD] and limit of quantitation [LOQ] of calibration, Figures S1 and 4, see Scheme 2). This new profile describes the evolving mass balance of UPLC-observable compounds during **1c**/H_2_O_2_ degradation of propranolol according to Scheme 2. The total molar concentration of all compounds included in Figure 4 at 5 min is 2.84 × 10^−5^ M, which corresponds to 61% of the initial propranolol. Therefore, the UPLC quantitation of peaks 2‒4 (Figure 2, tiny peak 1 could not be quantified because of crowding) is not providing a complete picture of the fate of the propranolol “naphthalene” unit. It is thus important to consider first other naphthalene-containing intermediates in an attempt to rationalize the fate of the unassigned 39 molar % of the naphthalene unit at 5 min. The intermediate 1-naphthol **A** was qualitatively detected by GC-MS. **A** was shown by kinetic analysis (see below and Table 1) to be degraded very quickly by **1c**/H_2_O_2._ The simulation of the degradation process described below suggests that **A** is an important contributor to the missing naphthalene unit at 5 min. In the process simulation described below, **A** appears as an important actor until all the propranolol is gone, forming first from propranolol and then degrading quickly. 1,4-dihydroxynaphthalene **B** is a common intermediate separating 1-naphthol **A** and 1,4-naphthoquinone **C** (Cason, 2004; Takata et al., 1983). However, **B** was not observed. In separate experiments, **B** was found to be so oxidatively sensitive that its mere dissolution in methanol, water, or acetonitrile, without added oxidant or **1c**, resulted in its conversion within seconds to **C**. The reaction is so fast that its rate could not be reliably measured by conventional techniques and spiking experiments were impossible to apply. Therefore, **B** is not a resident degradation product where toxicity needs to be considered, but instead a fast transforming intermediate to observable **C**. A good portion of the undetected total molar concentration is likely to be associated with the small ∗ peaks of unknown origin in Figure 2.

**Table 1:** Experimental and theoretical rate constants (both **1c**-catalyzed and uncatalyzed H_2_O_2_ oxidations) and zebrafish (embryo or larvae) lethality data for propranolol and its intermediates

Compound	*k*_I_	10^−2^×*k*_II_	10^−2^×*k*_II,S_	*k*_2_	*k*_2,S_	LC_50_/mg L^−1^
Propranolol^a^	90 ± 10	146 ± 2	170			2.48 (Sun et al., 2014)e)
1-Naphthol **A**	112 ± 7	500 ± 200	930			7.4930 (de Sousa Andrade, 2015)f)
1,4-Dihydroxynaphthalene **B**					3.5×10^3^	
1,4-Naphthoquinone **C**^b^			35			
1,4-Naphthoquinone **C**^c^				(36 ± 2)×10^−3^	34 × 10^−3^	0.2686 (de Pinho, 2013)^g^
2,3-Dihydro-2,3-epoxy-naphthalene-1,4-dione **D**^d^	5.0 ± 0.7	0.40 ± 0.07	0.70			
2-Hydroxynaphthalene-1,4-dione **E**^d^	107 ± 2	200 ± 10	500			0.1079 (de Pinho, 2013)^h^

Experimentally measured **1c**/H_2_O_2_ (*k*_I_, *k*_II_) and theoretical (*k*_II,S_) rate constants for propranolol, 1-Naphthol **A**, 2,3-dihydro-2,3-epoxy-naphthalene-1,4-dione **D**, and 2-hydroxynaphthalene-1,4-dione **E**; experimental (*k*_2_) and theoretical (*k*_2,S_) second-order rate constants for H_2_O_2_ direct oxidation of 1,4-dihydroxynaphthalene **B** and 1,4-naphthoquinone **C**. All *k* values (M^−1^ s^−1^) at pH 7, 25°C. See also Figures S9 and S10.

a Data are from Ref. (Somasundar et al., 2018).

b **E** product.

c **D** product via Equation 2.

d Phthalic acid **F** product.

e Zebrafish larvae 96-h LC_50_.

f Zebrafish embryo 96 hpf (hours postfertilization) 96-h LC_50_.

g Value for 2-methyl-1,4-naphthoquinone (menadione, Figure S10); zebrafish embryo 80 hpf (chronic exposure, chemical addition at 4 hpf) ~ 76-h LC_50_.

h Value for 5-hydroxy-1,4-naphthoquinone (juglone, Figure S10); zebrafish embryo 80 hpf (chronic exposure, chemical addition at 4 hpf) ~ 76-h LC_50_.

### Kinetics of oxidation by H_2_O_2_ of products of propranolol degradation, uncatalyzed (C) and 1c-catalyzed (A, D, E)

This section of the work was undertaken to quantitatively simulate the data in Figure 4 so that “theoretical” rate constants used in the simulations could be compared with those measured experimentally. As with propranolol, **A**, **D,** and **E** are predominantly oxidized by H_2_O_2_ catalytically, and the initial rates of disappearance of each were measured as a function of [H_2_O_2_]. The resulting hyperbolic dependences (Figure S9) were fitted to Equation 1, which is consistent with the mechanism in Scheme 1—the corresponding rate constants *k*_I_ and *k*_II_ are collected in Table 1.

The dual behavior of 1,4-naphthoquinone **C** is worth noting. It is presumably oxidized by H_2_O_2_ through two parallel pathways leading to two different products. Direct oxidation of **C** by H_2_O_2_ affords the epoxide **D** via a second-order pathway (Equation 2). Interestingly, whereas no **E** was detected in this uncatalyzed case, it was always detected in TAML/H_2_O_2_ propranolol oxidations. Therefore, it was assumed that **E** is produced from **C** catalytically according the rate law in Equation 1.

(Equation 2) $$\frac{d\left[D\right]}{dt}=k_{2}\left[H_{2}O_{2}\right]\left[C\right]$$

### Computer simulation of the concentration versus time profiles for propranolol and its degradation fragments

The results presented above establish multiple key steps in the chain of events of the TAML/H_2_O_2_ degradation of propranolol. Scheme 2 illustrates only the transformations of the naphthalene unit of the drug after the aliphatic component has been liberated. It does not account for the fate of the aliphatic unit of propranolol. The compounds set in bold were quantified by UPLC. Armed with the Scheme 2 characterizations that are quantitative for **C**-**E**, qualitative for **A** and **F**, and postulated on reasonable mechanistic grounds for **B**, together with the Table 1 rate constants for **1c**/H_2_O_2_ (*k*_II_) and H_2_O_2_ degradations (*k*_2_) as described above, it became feasible to simulate using KinTek Explorer the substantive *naphthalene unit* sub-story of the **1c**-catalyzed oxidation of propranolol.

**Figure 5:**
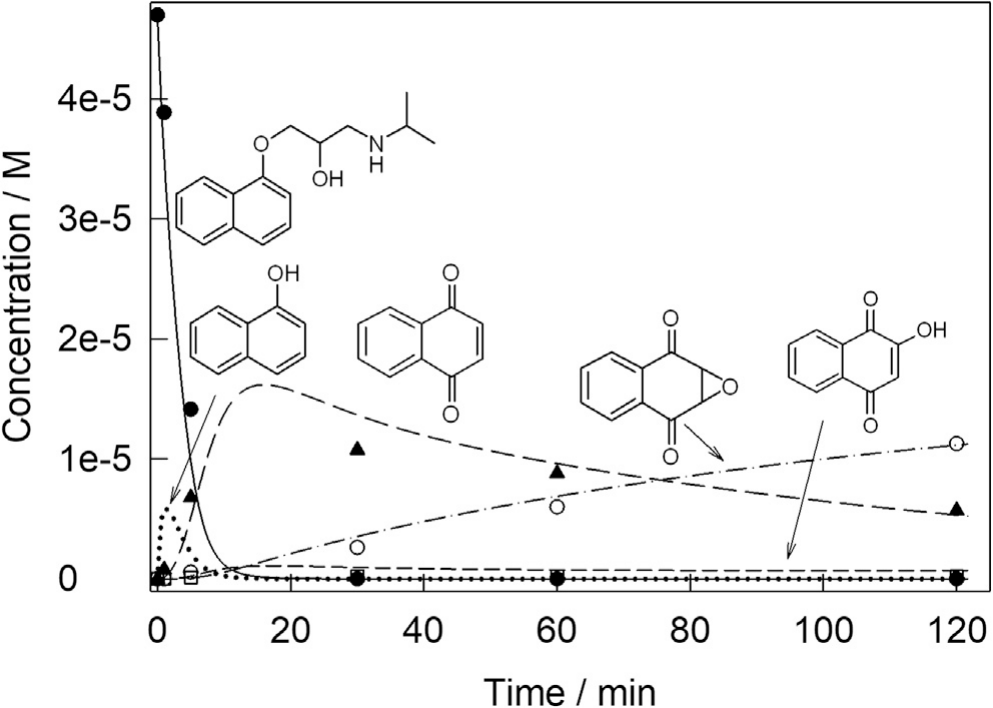
Simulated and experimental mass profiles for propranolol and its intermediates Simulated and experimental (UPLC) concentration versus time profiles for propranolol and its quantifiable fragment degradation processes (**C**-**E**) and unquantified 1-naphthol **A** (simulated including known *k*_II_, see text) during **1c**/H_2_O_2_ oxidation of propranolol. Lines were calculated using rate constants *k*_II,S_ estimated using KinTek software to attain the best visual match with the experimental data points. The operational instability of **1c** (Equation 3) with *k*_i_ of 1.7 × 10^−3^ s^−1^ was included in the KinTek simulation. See legend to Figure 4 for conditions and text for more details.

The TAML catalytic mechanism as in Scheme 1 was applied using the previously obtained rate constant *k*_I_ (90 M^−1^ s^−1^) measured with propranolol as the substrate, a known substrate-inhibited process (Somasundar et al., 2018). Experimentally, the *k*_I_ values for each of the fragment oxidations is similar except for **D** (Table 1). The simulation (Figure 5) was then derived by inputting *k*_II_ and *k*_2_ values for each step in the degradation process where these had been determined. The value of *k*_II_ was not available for 1,4-naphthoquinone **C** and therefore an arbitrary value of *k*_II_ was first input and then allowed to float freely. Preliminary efforts were made under the assumption that the catalytic activity of TAML catalyst **1c** *does not* change through the entire time span of Figure 4. The *k*_II_ and *k*_2_ values for propranolol and **A**-**E** were each slightly adjusted manually to obtain the best visual match between the experimental (UPLC) and simulated concentration versus time profiles for propranolol, **C, D,** and **E**. Using this approach, an acceptable visual match of the experimental and simulated results could not be obtained. However, much better agreement (Figure 5) was reached when the known inactivation (*k*_i_) of **1c** (operational instability) (Chanda et al., 2006; DeNardo et al., 2016; Emelianenko et al., 2014) was taken into account, i.e., step (*iii*) was added to the mechanism in Scheme 1. After visual optimization with *k*_i_ floating, the simulated value *k*_i,S_ = 1.7 × 10^−3^ s^−1^ was returned very close to the experimentally measured value of *k*_i_ = 1.1 × 10^−3^ s^−1^ that had been obtained for **1c** under the same conditions with Orange II as the substrate (DeNardo et al., 2016). The simulated lines in Figure 5 are calculated concentration versus time profiles using the catalytic rate constants *k*_II,S_ or the values of *k*_2,S_ when intermediates are directly oxidized by H_2_O_2_.

Active catalyst → Inactive catalyst → (*k*_i_) (Equation 3)

### Building calculated toxicity versus time profiles for propranolol and its degradation fragments

At first glance, the concentration versus time profiles in Figure 5 might convey the impression that the research goal of rendering propranolol harmless has been successfully achieved. The MP is rapidly destroyed, and its aromatic fragments, generated quickly in lower concentrations, all have disappeared by 8 h (Figure S1). However, total victory cannot be claimed because any degradation fragment(s) might also be toxic, even more toxic than propranolol, even at these low concentrations, and can have passing adverse impacts on the aquatic environment. Therefore, concentration profiles such as in Figure 5 should be accompanied by corresponding toxicity profiles in which concentrations are replaced by corresponding toxicities of preferably all compounds before their complete breakdown.

**Figure 6:**
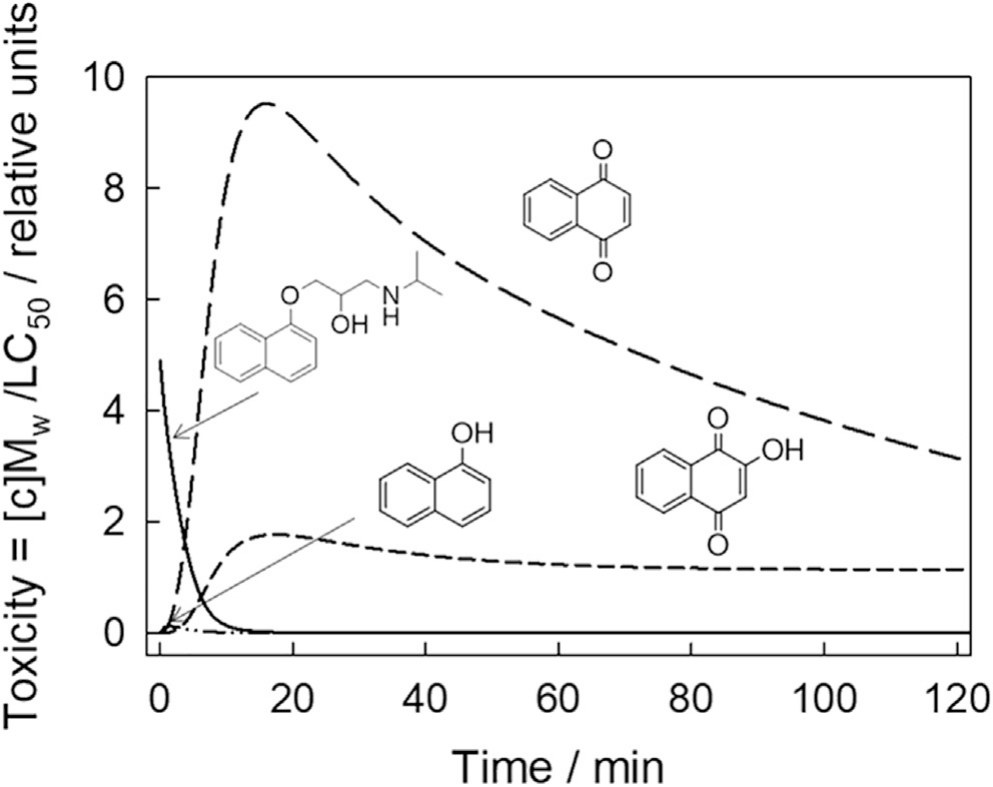
Calculated toxicity profiles for propranolol and its intermediates Calculated zebrafish embryos' toxicity profile from propranolol and its fragments during **1c**/H_2_O_2_ oxidation. See Figure 5 for the corresponding concentrations of propranolol and its fragments, the legend of Figure 5 for conditions, and the text for details. Zebrafish embryo toxicity values were calculated based on structural analogs for **C**, **D,** and **E** for which data are available. See Table 1 and Figure S10 for chemical structures of surrogates and further details.

However, for this approach to succeed, relevant toxicity data (Table 1) are required for at least a majority and preferably all the species in the decomposition profile (Figure 6). The toxicities of different chemicals to a single model species are different as are the toxicities of any single chemical to different models (EPA, n.d.). The toxicities further depend on the life stage of the model—embryo, adult, or other. Conditions of exposure are important because variables such as the solution pH affect toxicity (Bittner et al., 2018).

In 2013, a large team of endocrine disruption researchers and green chemists published their collective wisdom on which assays are suitable for detecting endocrine disruption (Schug et al., 2013). We have previously used the widely employed zebrafish model (embryo and adult) (McCollum et al., 2011) to assess the toxicity of TAML catalysts and processes (Onundi et al., 2017; Truong et al, 2013a, 2013b). No low-dose toxicity was observed for a member of the latest generation of TAMLs tested via the mouse uterotrophic assay (Warner et al., 2019). Zebrafish embryo developmental studies of seven TAML activators each at seven concentrations (80 nM–250 μM) showed that four TAMLs elicited no adverse effects; two showed several disruption endpoints at the two highest concentrations, 50 and 250 μM; and one killed all the fish at these two highest concentrations and disrupted multiple developmental endpoints at 10 μM—all seven catalysts produced no observable effects below 10 μM (Truong et al., 2013a). TAML/H_2_O_2_ eradicates adult fish feminization produced by the reproductive pill estrogen, ethinylestradiol, EE2 (Mills et al., 2015). Receptor activation (Ellis et al., 2009) and bacterial assays (Chen et al., 2012; Onundi et al., 2017) showed no low-dose adverse effects for the studied TAMLs. We plan to do many more EDC screening assays for any catalyst that might proceed to commercial development. Although the Environmental Protection Agency (EPA) database is colossal and there are some publications for propranolol toxicity to zebrafish, a complete set of comparable zebrafish data (all embryo or all adult, same conditions) even for the simplest of all endpoints, lethality (LC_50_), is not yet available. A full zebrafish toxicity analysis was outside the scope of this contribution to chemical methods development for assessing the sustainability critical environmental performance of TAML/H_2_O_2_ water purification (Warner et al., 2019), but the importance of expanding the study in this direction is explained below.

In the absence of ideal toxicity data, SciFinder and Google Scholar searches were performed to find the simplest 96-h LC_50_ (lethal concentration, 50%), values for the compounds of Scheme 2 with partial success. LC_50_ values (Table 1) were found for propranolol (larvae) (Sun et al., 2014), and 1-naphthol **A** (embryo) (de Sousa Andrade, 2015). The toxicity of 1,4-dihydroxynaphthalene **B** was considered to be of low importance because its high reactivity means it can only be fleetingly present in very low concentrations at any time in the TAML/H_2_O_2_ operating system. No data are available for 1,4-naphthoquinone **C**, 2,3-dihydro-2,3-epoxy-naphthalene-1,4-dione **D**, and 2-hydroxynaphthalene-1,4-dione **E**. Therefore, the available ~76-h LC_50_ (embryo) for 2-methyl-1,4-naphthoquinone (menadione) (de Pinho, 2013) and 5-hydroxynaphthalene-1,4-dione (juglone) (de Pinho, 2013) were used as surrogates for **C** and **E**, respectively.

The Figure 6 toxicity versus time profiles were calculated using the concentration data in Figure 5 and the LC_50_ values in Table 1. Figure 5 molar concentrations were converted to mg L^−1^ and then divided by the appropriate LC_50_ values. The toxicities in Figure 6 are thus normalized values at given concentrations. Although the calculated toxicity profile is incomplete, because only four of the seven known participants in Scheme 2 were taken into account among which two required the use of data-available surrogates; its potential significance is obvious from Figure 6 and validates the conclusion that this technique can be used extensively and powerfully when coupled to simultaneous toxicity testing.

The concentration of a targeted MP, propranolol, could go down, whereas zebrafish toxicity could increase as a result of the individual toxicity contributions from the evolving concentration changes of degradation fragments such as **C** and **E** in the degradation mixture. Fortunately, the determined increase is collectively temporary because none of the above degradation intermediates of Scheme 2 are detectable after 8 h of **1c**/H_2_O_2_/treatment (Figure S1). Compound **1c** is typically the most effective of the TAML catalyst family. However, the currently most effective NewTAML is more aggressive and approximately 10-fold faster in degradation processes (Warner et al., 2019) such that times to MP near-mineralization are anticipated to shorten considerably as NewTAMLs take their preeminent places in TAML/H_2_O_2_ processes.

It is also worth noting that the environmental situation is likely to be more complicated because profiles as in Figure 6 are made under the assumption that toxicity is proportional to the concentrations and toxicities of the individual components in a complex mixture. Summing toxicity contributions for each at any point in time during the degradation process may not accurately predict low-dose adverse effects, the nonlinear relationships between dose and response, which have been most vividly revealed for endocrine-disrupting chemicals (Vandenberg et al., 2012). This actuality will simply make the further development of the presented approach more informative and interesting.

### Real-world implications of the mass/toxicity profiling method

The method introduced above by the example of **1c**/H_2_O_2_ propranolol degradation provides a tool for assessing the presence and significance of toxic by-products in the degradation of MPs in water. As part of building a sustainable chemical enterprise, such degradation processes will need to be carried out on a planetary scale, and the more we understand how to assess the processes we might deploy for low-dose toxicity, the safer the future world will be. Coupling the calculated evolving toxicity profiles with the experimentally measured toxicities (full suite of assays that are sensitive to low-concentration adverse effects [locafs], Warner et al., 2019, and nonmonotonic dose/response toxicity), it should be possible to obtain a valid assessment of the dynamics of the impacts of TAML/H_2_O_2_ on water purification as the processes proceed. The method allows for examining (1) how and why the toxicity changes throughout a degradation process and (2) whether mixtures of toxic compounds contribute linearly or otherwise to the overall toxicity. The observation of no difference between the measured cumulative toxicity and the simulated toxicity would be evidence for the lack of synergistic effects, and the discovery of large differences would be evidence for non-linear mixture effects. As MPs are present in environmental waters at ppt-ppb concentrations, all the toxicity assays would need to be performed at similar concentrations and below to detect and quantify low-concentration adverse effects (locafs) caused by the MPs, their oxidation products, and the process medium.

### Mechanistic considerations

**Scheme 3:**
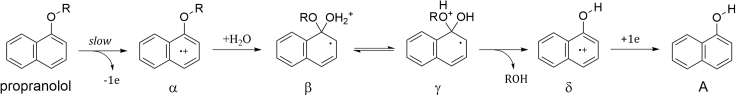
Postulated mechanism for conversion of propranolol to 1-naphthol A Postulated mechanism of oxidatively induced hydrolysis of propranolol to 1-naphthol during **1c**-catalyzed oxidative degradation of former by H_2_O_2_.

As postulated by several groups (Anquandah et al., 2013; Ganiyu et al., 2017; Isarain-Chavez et al., 2011), a key initial step in degradation of propranolol is its conversion to 1-naphthol **A** as a result of a cleavage of either the O–C_aryl_ or O–C_alkyl_ bond. Note that the nature of this step has never been mechanistically discussed in any detail. Propranolol, as in this study, is crashed oxidatively, although formation of 1-naphthol **A** is a hydrolytic non-redox substitution reaction, in which hydroxide is a formal incoming group. TAMLs catalyze the reaction at pH 7 wherein the concentration of OH^−^ is extremely low. Water as a nucleophile is insufficiently reactive toward propranolol—otherwise the drug should be degraded in pure water alone. Our data reported here and previously (Somasundar et al., 2018) show (see also Scheme 1) that the rate of propranolol degradation is limited by its interaction with the oxidized TAML-(active catalyst), which is presumably involved in a one-electron transfer (Collins and Ryabov, 2017) opening a door for succeeding hydrolytic transformations. The naphthalene ring of propranolol is a plausible target for electron abstraction. If this is the case, electron transfer from propranolol at presumably TAML iron(V)oxo intermediate (Collins and Ryabov, 2017) should afford a radical-cation α (Scheme 3), which might be more electrophilic than propranolol itself. Hypothetically, the radical-cation **α** may be subject to ipso attack by H_2_O to afford **β**. The tautomeric equilibrium should create a better leaving group ROH within **γ**. 1-Naphthol **A** is finally produced after one electron transfer at **δ**; iron(III) in the resting state of TAML could be a plausible electron donor.

Formally, the mechanism in Scheme 3 is oxidatively assisted (ether) hydrolysis. Provided “ether” is ignored, the term is not entirely new and has previously been used in connection with hydrolysis of allylic iodides (Yamamoto et al., 1983). Applied to nucleophilic substitution in aromatic series, first Alder (1980) and later Eberson (1983) used alternative, S_ON_2 terminology (nucleophilic substitution catalyzed by an oxidant), which corresponds exactly to the events in Scheme 3. In all cases, the oxidant increases electrophilicity of the substrate due to abstraction of an electron such that subsequent nucleophilic attack becomes more feasible.

### Conclusion

Detailed analytical and kinetic studies have made it possible to quantify the temporal behavior of many of the aromatic products during **1c**/H_2_O_2_ degradation of propranolol at 25°C and neutral pH. The data confirm that the decomposition pathway shown in Scheme 2 is by far the dominant degradation route and that minerals and near-minerals are the final products. There is now such a wide range of TAML activities (DeNardo et al., 2016) that the rates of transitions from one degradation intermediate to another can be controlled by the choice of the TAML, facilitating the detection of even quite reactive intermediates. Catalyst **1c** is about one-tenth as active as the most advanced NewTAML catalyst (Warner et al., 2019). Observing the NewTAML process intermediates would be all the more difficult because of the increased rates, which made **1c** an ideal catalyst for this study. Accurate time-dependent mass and approximate time-dependent acute toxicity profiles have been constructed revealing that the substantial decrease in the concentration of **1c**/H_2_O_2_-targeted propranolol and its degradation products does not guarantee environmental safety until all of the aromatic degradation fragments have been destroyed. As TAML/H_2_O_2_ typically makes laboratory assessment of the course of MP degradations possible, this contribution establishes a method for developing complete information on the potential environmental effects of complete decontamination procedures whereby mixtures can also be studied. Because TAML/H_2_O_2_ represents an accelerated form of the very metabolism that nature uses as its principal organic contaminant decomposition machinery, what is found in the laboratory is likely to be of direct environmental relevance.

In its totality, the study emphasizes a new that to understand in depth the potential ecological impacts of the TAML/peroxide oxidative degradation of propranolol, it is insufficient to simply remove the starting drug. Instead, critical understanding for the safety of an oxidative water purification technology encompasses (1) identification of as many as possible of the MP degradation intermediates, (2) building the concentration (mass) versus time profiles for MP and intermediates through kinetics with experimental and theoretically simulated data, (3) building relevant toxicity profiles from the mass profiles to understand how toxicity changes in the course of the oxidative degradation process, and, although not covered herein, (4) an exploration looking for changed outcomes when mixtures of MPs are present. Of particular future real-world value, this information can be used to guide the setting of process conditions to ensure the elimination of all toxic components.

### Limitations of the study

TAML/H_2_O_2_ provides a quick and easy MP decontamination technology for purifying water where significant toxicity studies point to the processes not introducing new forms of low-concentration toxicity. The systematic approach detailed in this work led us to the development of a simple method/tool to track the evolution of toxicity in the course of a multistep degradation of an MP in water. However, the toxicity model used is far from ideal. We outlined the applicability of the method utilizing zebrafish LC_50_ results cobbled together from multiple publications as this was best way to get a set of data. This highlights a vast and well-known data gap for aquatic toxicity in general. The toxicity profile would be much more relevant if founded on developmental assays of a model such as zebrafish all performed in the same laboratory under environmental conditions. Such a study should include investigations of multigenerational developmental toxicity. It was outside the scope of this work to perform such a study, but now that the methodology is established, this is a fruitful direction for further work. Moreover, the method should be able to detect the presence or lack of synergistic effects in the temporal toxicity profiles of degrading mixtures of MPs.

### Resource availability

#### Lead contact

Further information and requests for resources and reagents should be directed to and will be fulfilled by the Lead Contact, Terrence J. Collins (tc1u@andrew.cmu.edu).

#### Materials availability

This study did not generate new unique reagents.

#### Data and code availability

The published article includes all data generated or analyzed during this study.

## Methods

All methods can be found in the accompanying Transparent Methods supplemental file.

## Supplemental information

Document S1. Transparent Methods and Figures S1–S10

## References

[bib1] Alder R.W. (1980). Electron-transfer chain catalysis of substitution reactions. J. Chem. Soc., Chem. Commun..

[bib2] Anquandah G.A.K., Sharma V.K., Panditi V.R., Gardinali P.R., Kim H., Oturan M.A. (2013). Ferrate(VI) oxidation of propranolol: kinetics and products. Chemosphere.

[bib3] Benner J., Ternes T.A. (2009). Ozonation of propranolol: formation of oxidation products. Environ. Sci. Technol..

[bib4] Bittner L., Teixido E., Seiwert B., Escher B.I., Klüver N. (2018). Influence of pH on the uptake and toxicity of β-blockers in embryos of zebrafish, Danio rerio. Aquat. Toxicol..

[bib5] Carson R. (2002). Silent Spring.

[bib6] Cason J. (2004). Synthesis of benzoquinones by oxidation. Org. React..

[bib7] Chahbane N., Popescu D.L., Mitchell D.A., Chanda A., Lenoir D., Ryabov A.D., Schramm K.W., Collins T.J. (2007). FeIII-TAML-Catalyzed green oxidative degradation of the Azo dye Orange II by H2O2 and organic peroxides: products, toxicity, kinetics, and mechanisms. Green. Chem..

[bib8] Chanda A., Ryabov A.D., Mondal S., Alexandrova L., Ghosh A., Hangun-Balkir Y., Horwitz C.P., Collins T.J. (2006). Activity-stability parameterization of homogeneous green oxidation catalysts. Chemistry.

[bib9] Chen J.L., Ravindran S., Swift S., Wright L.J., Singhal N. (2012). Catalytic oxidative degradation of 17α-ethinylestradiol by FeIII-TAML/H2O2: estrogenicities of the products of partial, and extensive oxidation. Water Res..

[bib10] Collins T.J. (2002). TAML oxidant Activators:  A new approach to the activation of hydrogen peroxide for environmentally significant problems. Acc. Chem. Res..

[bib11] Collins T.J., Khetan S.K., Ryabov A.D., Anastas P.T. (2010). Handbook of green chemistry. Handbook of Green Chemistry.

[bib12] Collins T.J., Ryabov A.D. (2017). Targeting of high-valent iron-TAML activators at hydrocarbons and beyond. Chem. Rev..

[bib14] de Pinho B.R. (2013). Naphthoquinones and Ubiquinone Analogues Biological Properties: Modulation of Immune and Neurological Systems, PhD Thesis. https://search.proquest.com/docview/1927892336?accountid=26642%0Ahttp://link.periodicos.capes.gov.br/sfxlcl41?url_ver=Z39.88-2004&rft_val_fmt=info:ofi/fmt:kev:mtx:dissertation&genre=dissertations+%26+theses&sid=ProQ:ProQuest+Dissertations+%26+Theses+Global&.

[bib15] de Sousa Andrade T. (2015). Effects of Environmental Factors on the Toxicity of Pesticides to Zebrafish Embryos, PhD Thesis.

[bib16] DeNardo M.A., Mills M.R., Ryabov A.D., Collins T.J. (2016). Unifying evaluation of the technical performances of iron-tetra-amido macrocyclic ligand oxidation catalysts. J. Am. Chem. Soc..

[bib17] Eberson L. (1983). Catalysis by electron transfer in organic chemistry. J. Mol. Catal..

[bib18] Ellis W.C., Tran C.T., Denardo M.A., Fischer A., Ryabov A.D., Collins T.J. (2009). Design of more powerful iron-TAML peroxidase enzyme mimics. J. Am. Chem. Soc..

[bib20] Emelianenko M., Torrejon D., DeNardo M.A., Socolofsky A.K., Ryabov A.D., Collins T.J. (2014). Estimation of rate constants in nonlinear reactions involving chemical inactivation of oxidation catalysts. J. Math. Chem..

[bib21] EPA Ecotox Knowledgebase [WWW Document]. https://cfpub.epa.gov/ecotox/.

[bib22] Ganiyu S.O., Oturan N., Raffy S., Esposito G., Van Hullebusch E.D., Cretin M., Oturan M.A. (2017). Use of sub-stoichiometric titanium oxide as a ceramic electrode in anodic oxidation and electro-Fenton degradation of the beta-blocker propranolol: degradation kinetics and mineralization pathway. Electrochim. Acta.

[bib25] Ghosh A., Mitchell D.A., Chanda A., Ryabov A.D., Popescu D.L., Upham E.C., Collins G.J., Collins T.J. (2008). Catalase-peroxidase activity of iron(III)-TAML activators of hydrogen peroxide. J. Am. Chem. Soc..

[bib26] Gottlieb H.E., Kotlyar V., Nudelman A. (1997). NMR chemical shifts of common laboratory solvents as trace impurities. J. Org. Chem..

[bib27] Isarain-Chavez E., Cabot P.L., Centellas F., Rodriguez R.M., Arias C., Garrido J.A., Brillas E.L.B.-I.-C. (2011). Electro-Fenton and photoelctro-Fenton degradations of the drug beta-blocker propranolol using a Pt anode: identification and evolution of oxidation products. J. Hazard. Mater..

[bib29] Keelan P. (1965). Double-blind trail to propranolol (inderal) in Angina pectoris. Br. Med. J..

[bib30] Kundu S., Chanda A., Khetan S.K., Ryabov A.D., Collins T.J. (2013). TAML activator/peroxide-catalyzed facile oxidative degradation of the persistent explosives trinitrotoluene and trinitrobenzene in micellar solutions. Env. Sci. Technol..

[bib31] McCollum C.W., Ducharme N.A., Bondesson M., Gustafsson J. (2011). Developmental toxicity screening in zebrafish. Birth Defects Res. C Embryo Today.

[bib32] Mills M.R., Arias-Salazar K., Baynes A., Shen L.Q., Churchley J., Beresford N., Gayathri C., Gil R.R., Kanda R., Jobling S., Collins T.J. (2015). Removal of ecotoxicity of 17alpha-ethinylestradiol using TAML/peroxide water treatment. Sci. Rep..

[bib33] Nonoyama N., Oshima H., Shoda C., Suzuki H. (2001). The reaction of peroxynitrite with organic molecules bearing a biologically important functionality. The multiplicity of reaction modes as exemplified by hydroxylation, nitration, nitrosation, dealkylation, oxygenation, and oxidative dimerization and clea. Bull. Chem. Soc. Jpn..

[bib34] Onundi Y., Drake B.A., Malecky R.T., DeNardo M.A., Mills M.R., Kundu S., Ryabov A.D., Beach E.S., Horwitz C.P., Simonich M.T. (2017). A multidisciplinary investigation of the technical and environmental performances of TAML/peroxide elimination of Bisphenol A compounds from water. Green. Chem..

[bib36] Prichard B.N.C. (1964). Use of propranolol (Inderal) in treatment of hypertension. Br. Med. J..

[bib37] Quintana J.B., Rodil R., Cela R. (2012). Reaction of β-blockers and β-agonist pharmaceuticals with aqueous chlorine. Investigation of kinetics and by-products by liquid chromatography quadrupole time-of-flight mass spectrometry. Anal. Bioanal. Chem..

[bib38] Ryabov A.D., Collins T.J. (2009). Mechanistic considerations on the reactivity of green FeIII-TAML activators of peroxides. Adv. Inorg. Chem..

[bib39] Santiago-Morales J., Agüera A., Gómez M., Del M., Fernández-Alba A.R., Giménez J., Esplugas S., Rosal R. (2013). Transformation products and reaction kinetics in simulated solar light photocatalytic degradation of propranolol using Ce-doped TiO2. Appl. Catal. B Environ..

[bib40] Schug T.T., Abagyan R., Blumberg B., Collins T.J., Crews D., DeFur P.L., Dickerson S.M., Edwards T.M., Gore A.C., Guillette L.J. (2013). Designing endocrine disruption out of the next generation of chemicals. Green. Chem..

[bib41] Somasundar Y. (2020). On the Mechanism and Quantitative Toxicity Evolution of Catalytic TAML and NewTAML Hydrogen Peroxide Oxidative Destruction of Micropollutants in River Water and Municipal Wastewater.

[bib42] Somasundar Y., Lu I.C., Mills M.R., Qian L.Y., Olivares X., Ryabov A.D., Collins T.J. (2020). Oxidative catalysis by TAMLs: obtaining rate constants for non-Absorbing targets by UV-vis spectroscopy. ChemPhysChem.

[bib43] Somasundar Y., Shen L.Q., Hoane A.G., Tang L.L., Mills M.R., Burton A.E., Ryabov A.D., Collins T.J. (2018). Structural, mechanistic, and ultradilute catalysis portrayal of substrate inhibition in the TAML–hydrogen peroxide catalytic oxidation of the persistent drug and micropollutant. Propranolol. J. Am. Chem. Soc..

[bib44] Stapleton M.P. (1997). Sir James Black and propranolol. The role of the basic sciences in the history of cardiovascular pharmacology. Tex. Hear. Inst. J..

[bib45] Sun L., Xin L., Peng Z., Jin R., Jin Y., Qian H., Fu Z. (2014). Toxicity and enantiospecific differences of two β-blockers, propranolol and metoprolol, in the embryos and larvae of zebrafish (Danio rerio). Environ. Toxicol..

[bib46] Takata T., Tajima R., Ando W. (1983). Oxidation of dihydroxyaromatics by hypervalent iodine oxides: a facil quinone synthesis. J. Org. Chem..

[bib47] Tang L.L., DeNardo M.A., Gayathri C., Gil R.R., Kanda R., Collins T.J. (2016). TAML/H2O2 oxidative degradation of metaldehyde: pursuing better water treatment for the most persistent pollutants. Env. Sci. Technol..

[bib48] Truong L., DeNardo M.A., Kundu S., Collins T.J., Tanguay R.L. (2013). Zebrafish assays as developmental toxicity indicators in the green design of TAML oxidation catalysts. Green. Chem..

[bib49] Truong L., Reif D.M., St Mary L., Geier M.C., Truong H.D., Tanguay R.L. (2013). Multidimensional in vivo hazard assessment using zebrafish. Toxicol. Sci..

[bib50] Vandenberg L.N., Colborn T., Hayes T.B., Heindel J.J., Jacobs D.R., Lee D.H., Shioda T., Soto A.M., vom Saal F.S., Welshons W.V. (2012). Hormones and endocrine-disrupting chemicals: low-dose effects and nonmonotonic dose responses. Endocr. Rev..

[bib51] Warner G.R., Somasundar Y., Jansen K.C., Kaaret E.Z., Weng C., Burton A.E., Mills M.R., Shen L.Q., Ryabov A.D., Pros G. (2019). Bioinspired, multidisciplinary, iterative catalyst design creates the highest performance peroxidase mimics and the field of sustainable ultradilute oxidation catalysis (SUDOC). ACS Catal..

[bib52] Warner G.R., Somasundar Y., Weng C., Akin M.H., Ryabov A.D., Collins T.J. (2020). Zero-order catalysis in TAML-catalyzed oxidation of imidacloprid, a neonicotinoid pesticide. Chemistry.

[bib53] Yamamoto S., Itani H., Tsuji T., Nagata W. (1983). Oxidatively assisted hydrolysis of allylic iodides to rearranged allylic alcohols. A new example of [2, 3] sigmatropic rearrangement. J. Am. Chem. Soc..

