# Supplementary material for: Quantifying evolving toxicity in the TAML/peroxide mineralization of propranolol
Source: iScience. 2020 Dec 7;24(1):101897. doi: 10.1016/j.isci.2020.101897 (PMC7753967; doi:10.1016/j.isci.2020.101897)
Supplement: Document S1. Transparent Methods and Figures S1–S10 [file mmc1.pdf]

iScience, Volume *24*

Supplemental Information

Quantifying evolving toxicity in the TAML/peroxide mineralization of propranolol

Yogesh Somasundar, Abigail E. Burton, Matthew R. Mills, David Z. Zhang, Alexander D. Ryabov, and Terrence J. Collins

Supplemental Information

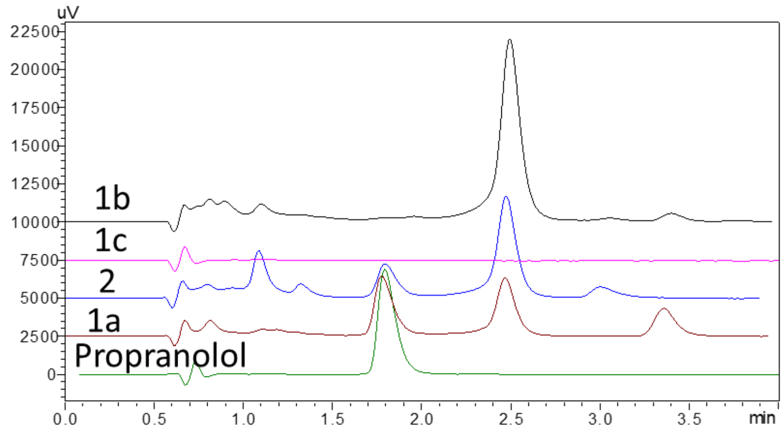

Top

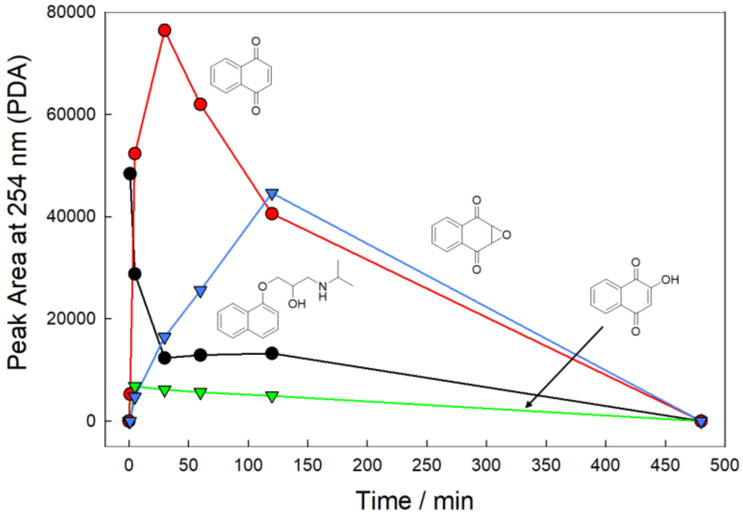

Bottom

Figure S1. **Related to Figure 2.** (Top) UPLC chromatograms for various TAML/H_2O_2 treatments for propranolol after 8h of reaction. (Bottom) Peak areas at 254 nm, using photo-diode array detector, of propranolol and peaks 2–4 in Figure 1 for **1c**-catalyzed degradation of propranolol by H_2O_2 after 8 h. Lines are for emphasis only. Calibration curves were developed for all compounds for PDA detection at 254 nm. Reaction conditions: [**1c**] = 1×10^{-6} M, [H_2O_2] = 5×10^{-3} M, [propranolol] = 50×10^{-6} M. 0.01 M phosphate buffer, pH 7, 25 °C. LOD and LOQ for UPLC quantifications:

MP or transformation product	LOD (M)	LOQ (M)
propranolol	5.8×10^{-7}	1.7×10^{-6}
1,4-naphthoquinone **C**	6.1×10^{-8}	1.8×10^{-7}
2,3-Dihydro-2,3-epoxy-naphthalene-1,4-dione **D**	5.1×10^{-7}	1.5×10^{-6}
2-Hydroxynaphthalene-1,4-dione **E**	1.4×10^{-7}	4.3×10^{-7}

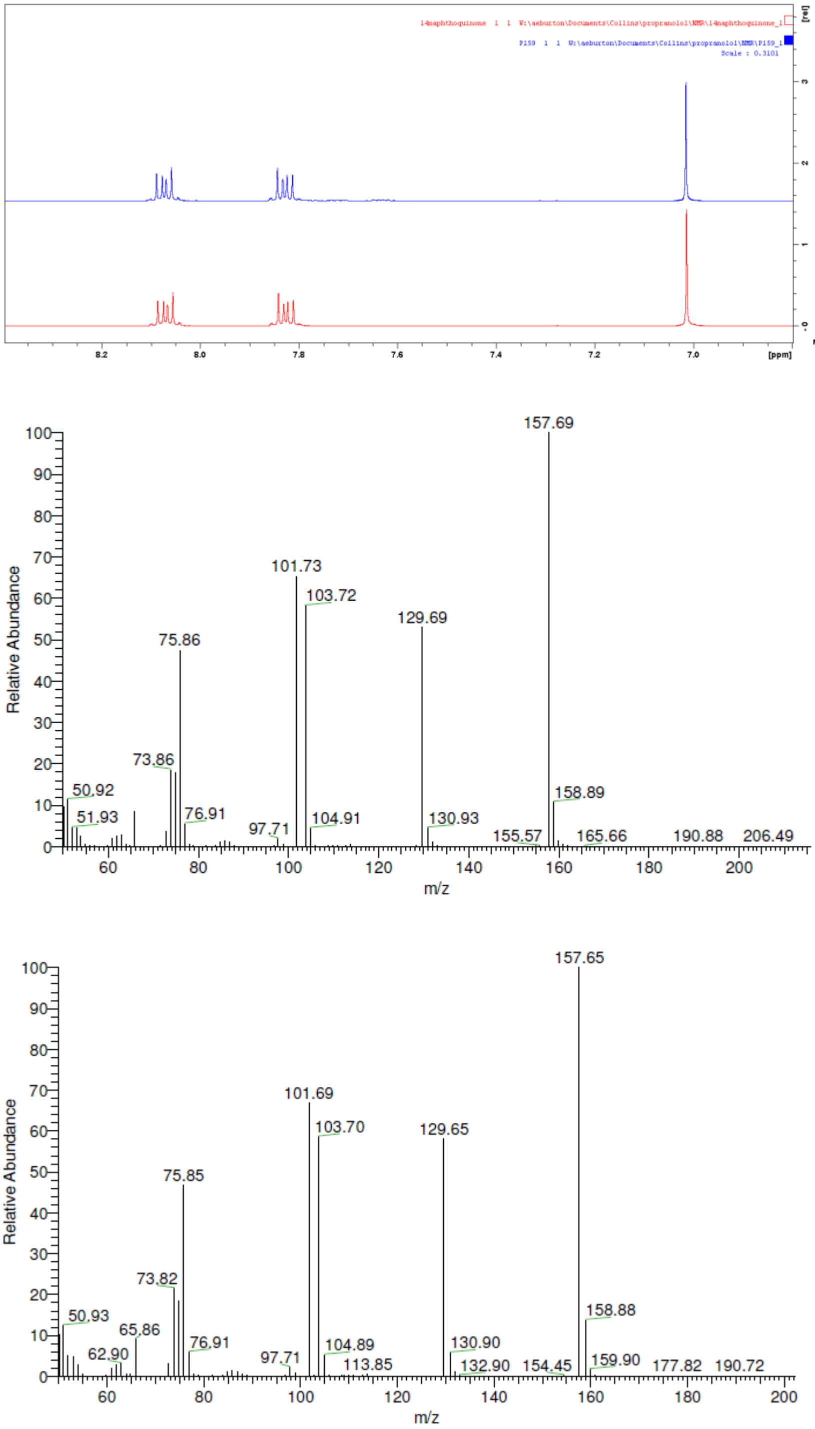

Top

Middle (made)

Bottom (standard)

Figure S2. **Related to Figure 2.** (Top) ^{1}H NMR spectra (in methanol-d4) of experimentally obtained (blue, top) and standard (red, bottom) 1,4-naphthoquinone.**C.** Mass spectra of 1,4-naphthoquinone **C** obtained from propranolol (Middle) and standard sample (Bottom) in positive ionization mode.

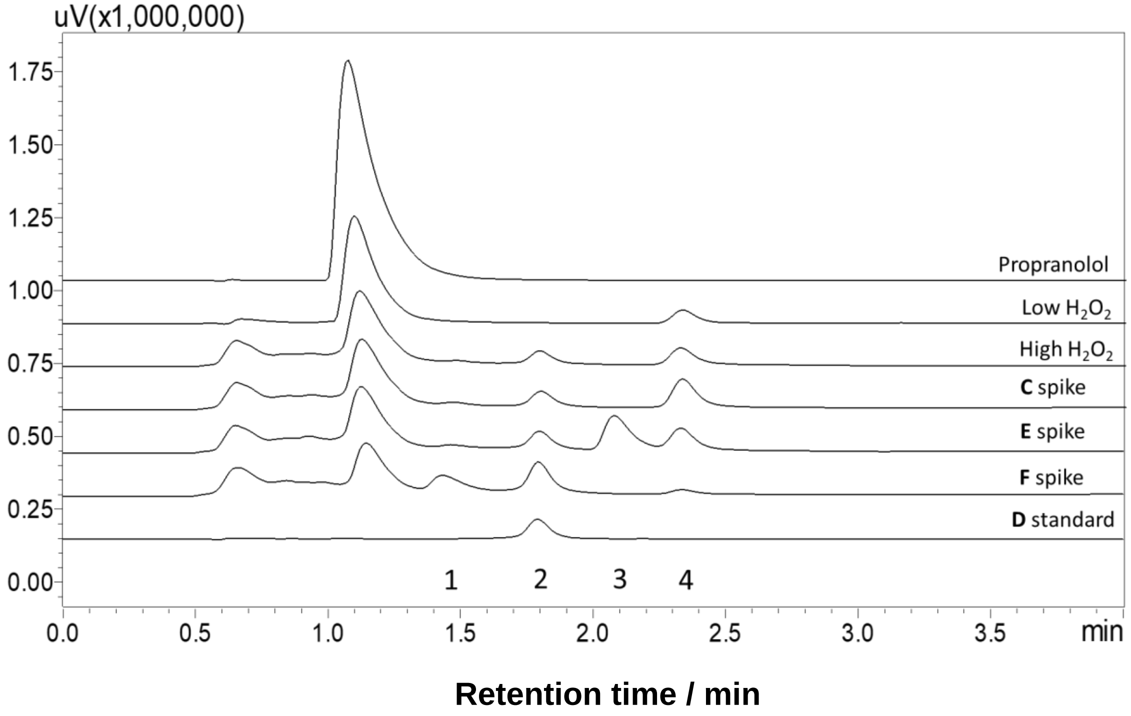

Figure S3. **Related to Figure 2.** UPLC chromatograms of propranolol oxidation by **1c**/ H_2O_2, (PDA 254 nm) under different reaction conditions and upon spiking with different chemical standards. Reaction conditions: [**1c**] = 1 × 10^{-6} M, [H_2O_2] = 5 × 10^{-4} M (low) and 5 × 10^{-3} M (high), [propranolol] = 50 × 10^{-6} M. 0.01 M phosphate buffer, pH 7, 25 °C. [Stock solutions of Intermediate standards] = 5 × 10^{-4} M.

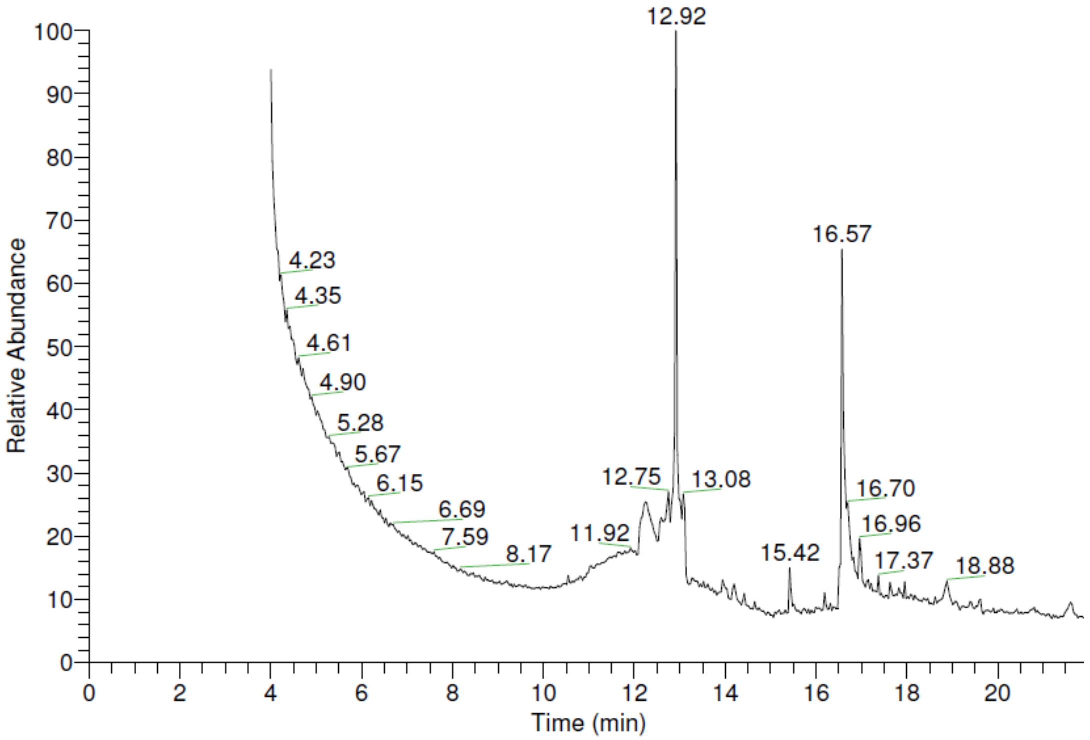

Figure S4. **Related to Figure 2.** Gas chromatogram of the products of **1c**/H_2O_2 propranolol oxidation after solid phase extraction into methanol. Conditions: [propranolol] = 1.7 ×10^{-3} M, [**1c**] = 1 ×10^{-6} M, $[H_2O_2]_{total}$ = 76 ×10^{-3} M, unbuffered HPLC water, 25 °C. Aliquots of H_2O_2 were added over 10 days. The aqueous mixture after 10 days was solid phase extracted into methanol. In this experiment a large amount of propranolol was decomposed with insufficient **1c** to complete the degradation as a method for capturing sizeable quantities of degradation intermediates. Experimental Section (instrumentation) for GC conditions.

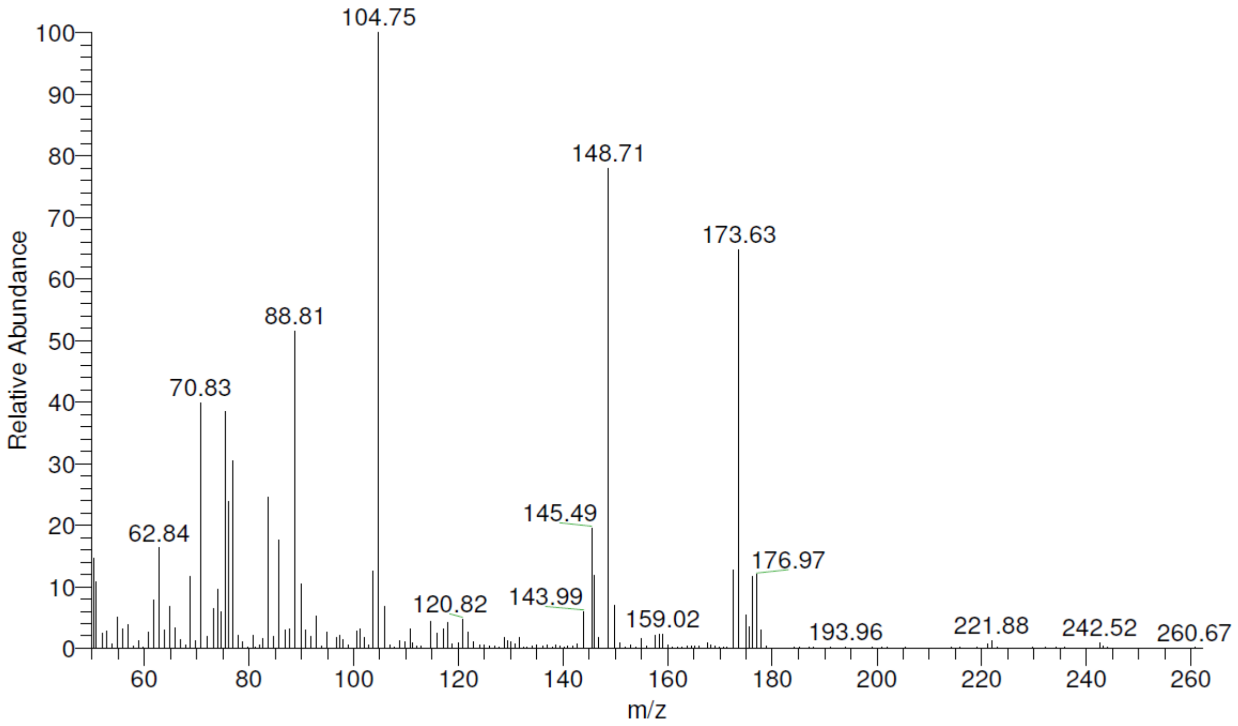

Figure S5. **Related to Figure 2.** EI-mass spectrum of the peak with GC retention time 12.92 min ascribed to 2,3-dihydro-2,3-epoxy-naphthalene-1,4-dione **D**. See Figure S4 for reaction conditions and Experimental Section (instrumentation) for MS conditions.

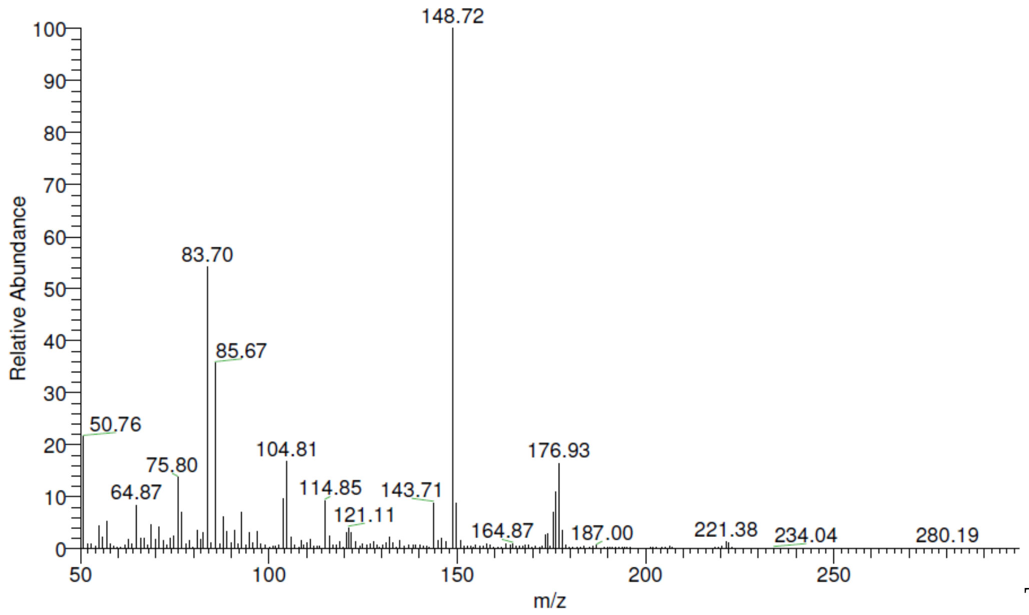

Top (observed)

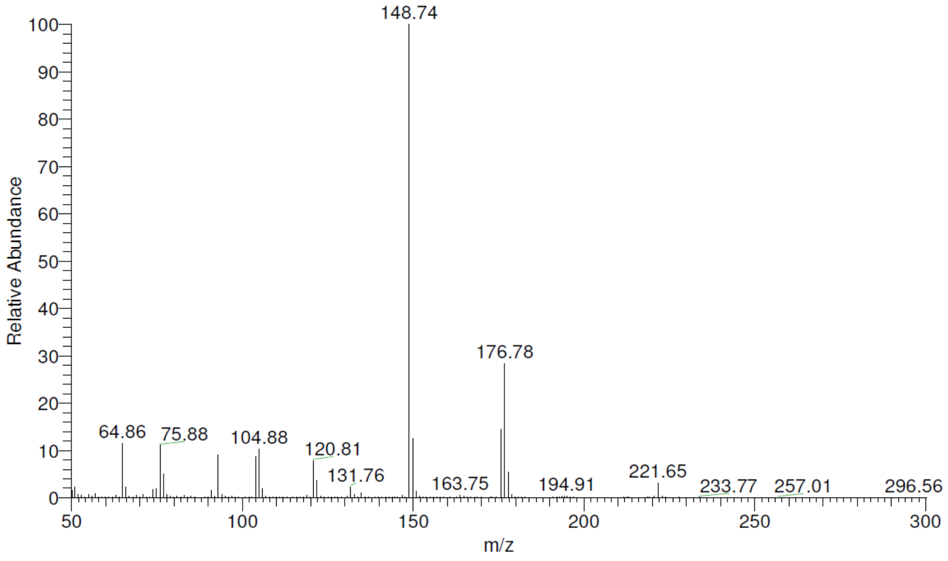

Bottom (standard)

Figure S6. **Related to Figure 2.** (Top) EI-mass spectrum of the peak with retention time 13.08 min ascribed to 2-hydroxynaphthalene-1,4-dione **E**. (Bottom) The spectrum of 2-hydroxynaphthalene-1,4-dione **E** standard. See Figure S4 for reaction conditions and Experimental Section (instrumentation) for MS conditions.

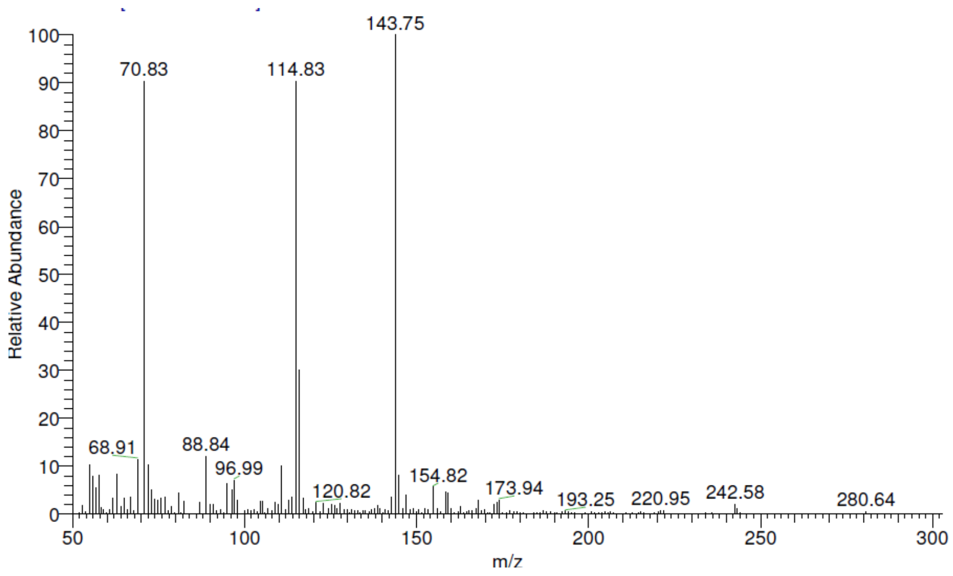

Top (observed)

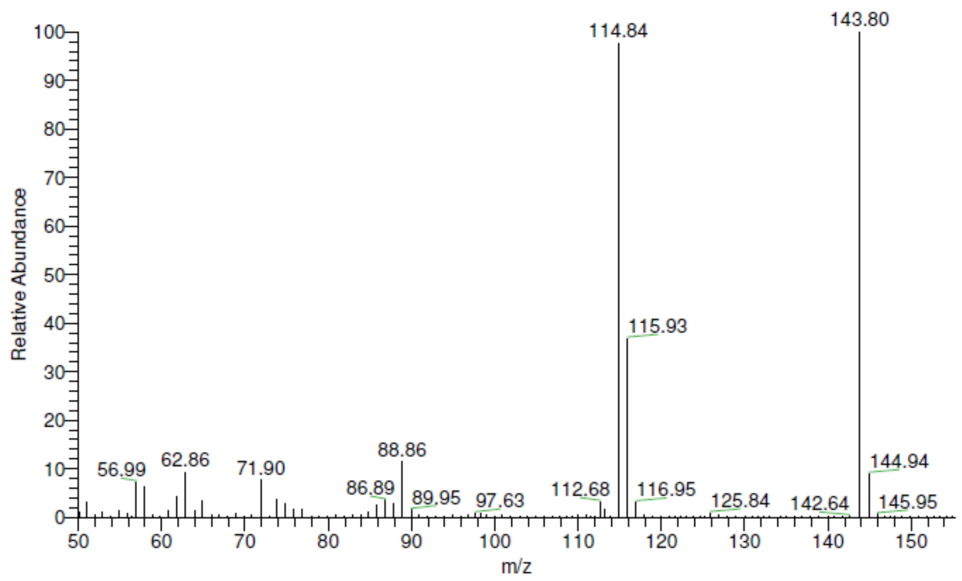

Bottom (standard)

Figure S7. **Related to Figure 2.** (**Top**) EI-mass spectrum of the peak with retention time 12.75 min ascribed to 1-naphthol **A**. (**Bottom**) The spectrum of 1-naphthol **A** standard. See Figure S4 for reaction conditions and Experimental Section (instrumentation) for MS conditions.

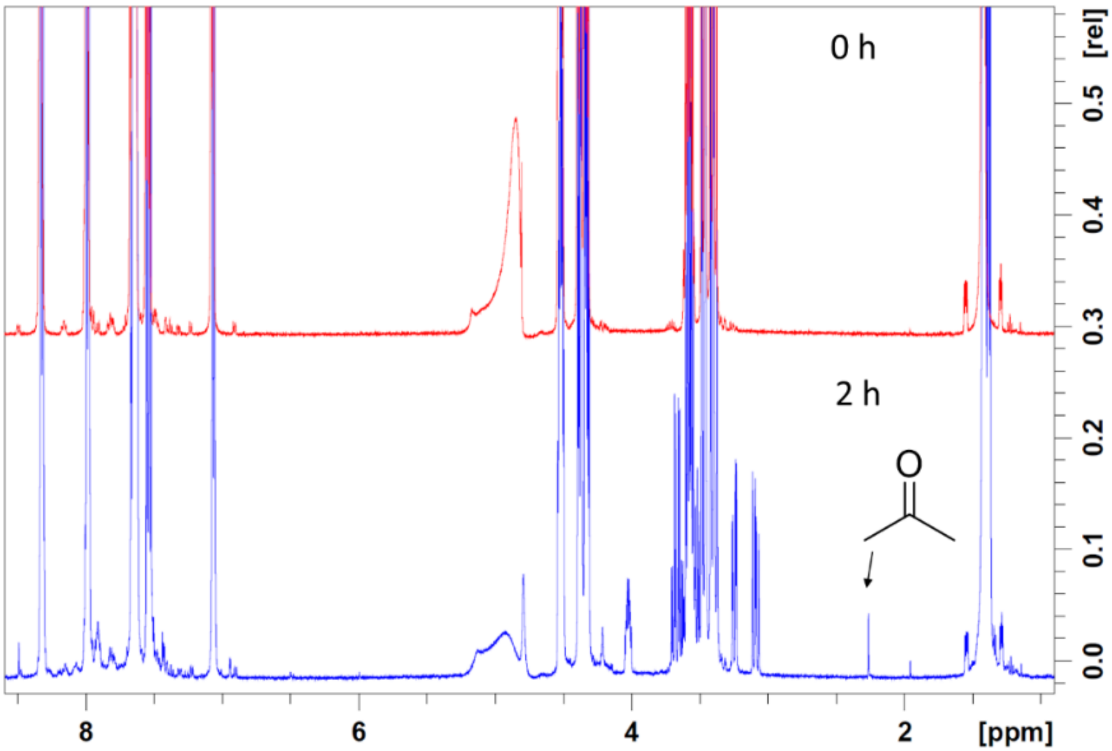

Figure S8. **Related to Figure 3.**^{1}H NMR spectra (in D_2O) of propranolol before (red, top) and after 2 h of **1c**/H_2O_2 oxidation (blue, bottom) Conditions: [**1c**] = 1 × 10^{-6} M, [propranolol] = 1.3 × 10^{-2} M, [H_2O_2] = 0.3 M, pH 7 (0.01 M phosphate in D_2O).

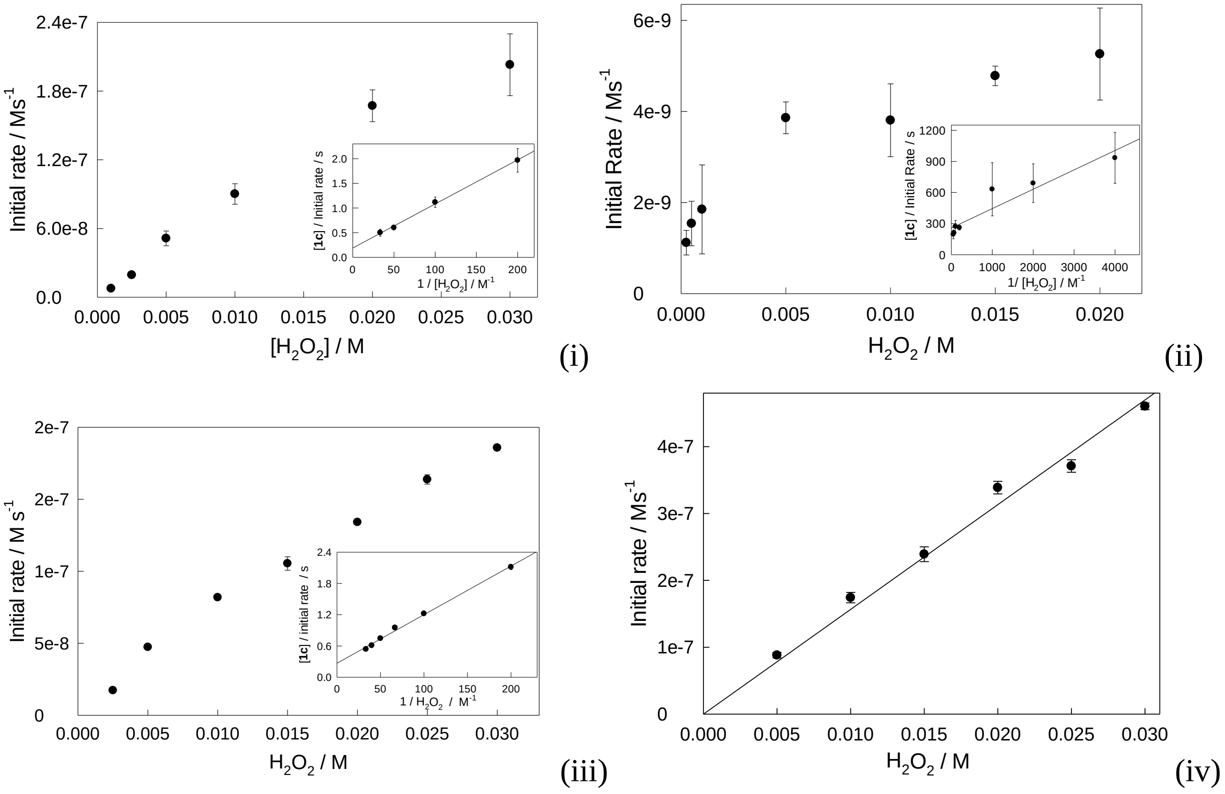

Figure S9. **Related to Table 1.** Initial rates versus [H_2O_2] for **1c**/H_2O_2 oxidative degradation of (i)1-naphthol **A**, (ii) 2,3-dihydro-2,3-epoxy-naphthalene-1,4-dione **D**, (iii) 2-hydroxynaphthalene-1,4-dione **E**. (iv) Initial rates versus [H_2O_2] for uncatalyzed oxidation by H_2O_2 of 1,4-naphthoquinone **C**. Insets to (i), (ii) and (iii) show the corresponding double inverse plots, [**1c**]/(initial rate) versus $[H_2O_2]^{-1}$.

General Reaction Conditions: pH 7 (0.01 M phosphate) and 25 °C.

Specific Conditions for (i): [**1c**] 1×10^{-7} M, [H_2O_2] (1-30) × 10^{-3} M, [**A**] 1.2×10^{-4} M

Specific Conditions for (ii): [**1c**] 1×10^{-6} M, [H_2O_2] (0.25-20) × 10^{-3} M, [**D**] 1.3×10^{-4} M

Specific Conditions for (iii): [**1c**] 1×10^{-7} M, [H_2O_2] (2.5-30) × 10^{-3} M, [**E**] 2×10^{-4} M

Specific Conditions for (iv): [H_2O_2] (5-30) × 10^{-3} M, [**C**] 4.3×10^{-4} M

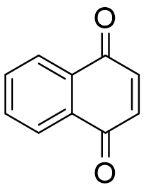

1,4-naphthoquinone

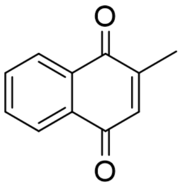

2-methyl-1,4-naphthoquinone (menadione)

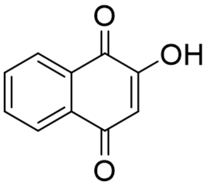

2-hydroxynaphthalene-1,4-dione

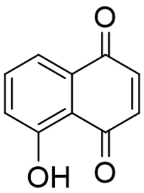

5-hydroxynaphthalene-1,4-dione (juglone)

Figure S10. **Related to Table 1 and Figure 6.** Structures of (left) 1,4-naphthoquinone **C**, 2-hydroxynaphthalene-1,4-dione **E**, and (right) respective surrogates, 2-methy-1,4-naphthoquinone (menadione), and 5-hydroxynaphthalene-1,4-dione (juglone) used for the acute toxicities calculations in Figure 5.

Transparent Methods

Materials

TAML activator **1a** was obtained from GreenOx Catalysts, Inc.; other activators were obtained from lab supplies.(Ellis et al., 2010; Ghosh, 2004; Popescu et al., 2008) Buffer solutions were made using KH_2PO_4 (Acros) or K_2HPO_4 (Merck) and the pH was adjusted with concentrated solutions of KOH or H_3PO_4. Hydrogen peroxide (30%) was purchased from Fischer. Catalase from bovine liver (lyophilized powder, 2000-5000 units mg^{-1} of protein) was purchased from Sigma. (±)-Propranolol hydrochloride (>99%), sodium nitrite, sodium nitrate, sodium acetate, phthalic acid dipotassium salt, sodium pyruvate, sodium oxalate, sodium oxamate, maleic acid disodium salt, and sodium chloride were purchased from Sigma and used as received. Methanol and water (both HPLC grade) were obtained from Fischer and used for the experimental degradations and for liquid or ion chromatography without additional purification.

Instrumentation

UV-vis measurements were performed using an Agilent 8453 instrument with an attached temperature controller. The pH measurements were made using an Accumet Basic AB15 pH meter from Fischer Scientific. UPLC studies were performed with a Shimadzu LC system with LC 20AB pump, SIL 20A autosampler, CTO 20A column oven, and an RF 20A XS fluorescence detector. A Kinetex (Phenomenex) 5 µM EVO C18 100A column (4.6 × 50 mm) was used for all kinetic analyses. The LC method consisted of 1 mL min^{-1} flow rate, 35% methanol in pH 3 phosphate buffer (0.01 M), 40 °C column temperature, and fluorescence detection with 230 nm excitation and 340 nm emission. The sample injection volume for the analyses was 10 µL and the data was automatically integrated and analyzed using Lab Solutions software for quantitation of the suite of compounds in the propranolol TAML/H_2O_2 degradation profile. Ion chromatography analyses were performed using the Thermoscientific Dionex Ion chromatography system (ICS) 5000+ with Dionex ICS-5000+ dual pump, Dionex ICS-5000+ eluent generator, Dionex ICS-5000+ detector/chromatography module housing a conductivity detector, and a Thermo Scientific Dionex AS-AP autosampler. A Dionex IonPac CG12 A 4 × 50 mm guard column and a Dionex AS11HC 4 × 250 mm column were used throughout. Analyses were performed under gradient conditions with concentration of KOH (mobile phase) being 1 (5 min), 15 (14 min), 30 (23 min) and 60 mM

(40 min). The IC method under gradient conditions comprised of 0.9 mL min^{-1} flow rate with an oven temperature of 30 °C and a 25 μL injection volume. The mobile phase was prepared with deionized water from a Barnstead Nanopure system. Data were analyzed using Chromeleon Chromatography Version 7 software. Gas chromatography-mass spectrometry analyses were performed with a Restek Rxi® -XLB (30 m, 0.25 mm ID, 0.25 µM) column using a Thermo Finnigan gas chromatograph (GC) equipped with a Trace DSQ mass spectrometer, a programmable temperature vaporization (PTV) injector, and a COMBI PAL autosampler (LEAP technologies, CTC Analystics). Helium was used as a carrier gas in a constant flow mode of 1 mL min^{-1}. Each GC-MS analysis took 22 min. The chromatographic oven temperature was 40 °C for the first 4 min and was increased to 300 °C thereafter at a speed of 20 °C min^{-1} till the 17th minute after which it was kept at 300 °C. An injection port temperature of 240 °C and a transfer line temperature of 300 °C were employed. The ion source was kept at 200 °C. The mass spectrometer was operated in the scan mode (scan range m/z 50-1000) and it was turned on after 4 min and ended at 22 min for each analysis. The electron ionization mode was applied (70 eV). All samples were analyzed in a positive ionization mode. The sample (10 µL) was injected in a split mode (split ratio 10). GC-MS data was processed with Xcalibur software. ^{1}H NMR data were collected at 25 °C on a Bruker Avance III 500 MHz spectrometer and processed with Bruker TopSpin 3.5 software.

Analyses for Propranolol Fragments

The multistep TAML-catalyzed hydrogen peroxide oxidation of propranolol was quantified by UPLC. A reaction mixture was prepared by adding aliquots of propranolol (5×10^{-3} M), TAML catalyst (5×10^{-3} M) to 0.01 M phosphate buffer and equilibrated to 25 °C in a water bath. A reaction was initiated by adding an aliquot of H_2O_2 (1 M) to the reaction mixture.(Somasundar et al., 2018) At suitable time intervals, aliquots of the reaction mixture were analyzed by UPLC, IC, GC-MS, and ^{1}H NMR. Intermediates and product ions were confirmed either by spiking standards to the reaction mixtures or by running a standard separately under similar conditions.

Kinetics of Oxidation of Propranolol Fragments

Kinetics studies of **1c**-catalyzed oxidation of 2,3-dihydro-2,3-epoxy-naphthalene-1,4-dione (*N*O_2epo) and 2-hydroxynaphthalene-1,4-dione (*N*O_2(OH)) were performed and followed by

UV/vis spectroscopy. Initial rates were determined by monitoring a decrease in absorbance at 275 ($\varepsilon = 5840\ M^{-1}\ cm^{-1}$) and 460 nm ($\varepsilon = 3000\ M^{-1}\ cm^{-1}$) for *N*$O_2$epo and *N*$O_2$(OH), respectively. The oxidation of 1-naphthol was followed by measuring absorbance accumulation at 520 nm(Das et al., 1998) where a 1-naphthol oxidation derivative gives a clearly separated absorbance for which the value of $\varepsilon = 3000\ M^{-1}\ cm^{-1}$ was experimentally determined—this compound is likely to be radical product of **B**, see below). The kinetics of the non-catalyzed oxidation of 1,4-naphthoquinone by H_2O_2 was monitored by a decrease in absorbance at 345 nm ($\varepsilon = 3320\ M^{-1}\ cm^{-1}$, experimentally determined). Reaction mixtures for intermediate oxidations were prepared by adding suitable amounts of stock solutions of the subject naphthalene derivative, **1c** (for catalyzed reactions) and phosphate buffer. Then, the reaction was initiated by adding H_2O_2. Runs were conducted at 25 °C in 0.01 M phosphate buffer. Stock solutions of **1c** (5×10^{-3} M), naphthalene derivative standards (5×10^{-3} M), and H_2O_2 (1 M) were prepared in HPLC grade water. Solutions of H_2O_2 were standardized by UV spectroscopy at 230 nm in water ($\varepsilon = 72.4\ M^{-1}\ cm^{-1}$).(George, 1953) All the simulations were developed using the software KinTek Explorer ver. 6.(Johnson et al., 2009)

Isolation of 1,4-Naphthoquinone

Degradation of propranolol was conducted first by dissolving 100 mg (0.34 mmol) propranolol hydrochloride in HPLC grade water (200 mL) at high concentration. An appropriate volume of **1c** stock solution was added to give a concentration of 1×10^{-6} M. Hydrogen peroxide solution was then added in multiple aliquots every ca. 24 h over 10 days (total amount added 15.2 mmol, 1.04 × mineralization requirement). 1,4-Naphthoquinone was then isolated by solid-phase extraction (SPE) of the aqueous degradation mixture into methanol (80 mL) and the solvent was removed by rotary evaporation. The brownish-red residue was dissolved in a minimum amount of ethanol and loaded onto a basic silica flash column. The elution was performed using a gradient method starting with 60:40:1 EtOAc:petroleum ether:NEt_3 progressing incrementally to the 50:50:1 ethanol: EtOAc:NEt_3. Yellow fractions containing 1,4-naphthoquinone were eluted first, combined and the solvent was removed by rotary evaporation. The yellow liquid obtained was further purified by flash column chromatography using a solvent gradient from 10:90 EtOAc:petroleum ether to 100% ethyl acetate. The solution of 1,4-naphthoquinone was dried in air and the product was dissolved in d_4-methanol and analyzed by ^{1}H NMR and GC-MS.

Computer Simulations

A computer simulation based on Kintek Explorer (Kintek simulates from the reaction scheme of an overall process with the rate constants of the individual steps as inputs) was then developed for the total **1c**/H_2O_2 propranolol degradation by (i) creating reaction Scheme 2 from the experimentally uncovered stepwise oxidative process of the naphthalene unit of the drug, (ii) measuring the k_{II} or k_2 independently by the initial rate method for **1c**/H_2O_2 degradation of each observable intermediate, (iii) fitting the overall experimentally determined disappearance of propranolol and each intermediate based on the competition of all these compounds for the TAML active intermediate (k_{II}) or H_2O_2 (k_2), (iv) making slight adjustments in the k_{II} and k_2 values of propranolol and all intermediates to obtain the best visual matches of the simulations with the experimentally determined loss of [propranolol] and each of the four trackable degradation intermediates and, (v) including in the simulations the experimentally known TAML inactivation rate constant k_i without which, as is to be expected,(DeNardo et al., 2016) good agreement between the simulated and experimental decays could not be reached.

References

Das, T.N., and Neta, P. (1998). Reduction potentials of naphthoxyl and pyridoxyl radicals in aqueous solutions. J. Phys. Chem. A *102*, 7081–7085.

DeNardo, M.A., Mills, M.R., Ryabov, A.D., and Collins, T.J. (2016). Unifying Evaluation of the Technical Performances of Iron-Tetra-amido Macrocyclic Ligand Oxidation Catalysts. J. Am. Chem. Soc. *138*, 2933–2936.

Ellis, W.C., Tran, C.T., Roy, R., Rusten, M., Fischer, A., Ryabov, A.D., Blumberg, B., and Collins, T.J. (2010). Designing Green Oxidation Catalysts for Purifying Environmental Waters. J. Am. Chem. Soc. *132*, 9774–9781.

George, P. (1953). The chemical nature of the second hydrogen peroxide compound formed by cytochrome c peroxidase and horseradish peroxidase. 1. Titration with reducing agents. Biochem. J. *54*, 267–276.

Ghosh, A. (2004). Design, Synthesis and Mechanistic Studies of Iron-TAML Catalytic Activators of Hydrogen Peroxide and a New Activation chemistry of Dioxygen by Iron. Phd Thesis. Carnegie Mellon University.

Johnson, K.A., Simpson, Z.B., and Blom, T. (2009). Global Kinetic Explorer: A new computer program for dynamic simulation and fitting of kinetic data. Anal. Biochem. *387*, 20–29.

Popescu, D.L., Chanda, A., Stadler, M.J., Mondai, S., Tehranchi, J., Ryabov, A.D., and Collins, T.J. (2008). Mechanistically inspired design of FeIII-TAML peroxide-activating catalysts. J. Am. Chem. Soc. *130*, 12260–12261.

Somasundar, Y., Shen, L.Q., Hoane, A.G., Tang, L.L., Mills, M.R., Burton, A.E., Ryabov, A.D., and Collins, T.J. (2018). Structural, Mechanistic, and Ultradilute Catalysis Portrayal of Substrate Inhibition in the TAML–Hydrogen Peroxide Catalytic Oxidation of the Persistent Drug and Micropollutant, Propranolol. J. Am. Chem. Soc. *140*, 12280–12289.
